# Organizational and financial challenges in care transitions: a qualitative study of long-term care systems in Germany, the Netherlands and Poland

**DOI:** 10.1186/s12877-025-06842-4

**Published:** 2025-12-11

**Authors:** Estera Wieczorek, Ewa Kocot, Silvia Evers, Christoph Sowada, Milena Pavlova

**Affiliations:** 1 Department of Health Economics and Social Security, Institute of Public Health, Faculty of Health Sciences, Jagiellonian University Medical College, Skawińska 8, Krakow, 31-066 Poland; 2 Department of Health Services Research, Care and Public Health Research Institute (CAPHRI), Faculty of Health, Medicine and Life Sciences, Maastricht University Medical Centre, Maastricht University, P.O. Box 616, Maastricht, 6200 MD The Netherlands

**Keywords:** Transitional care, Care transitions, Coordinated care, Older adults, Long-term care, Organizational, Financial, Qualitative

## Abstract

**Background:**

Suboptimal care transitions are common and have been linked to adverse events, hospital readmissions, low satisfaction with care, and high healthcare costs. Broader organizational and financial aspects are known to affect care transitions in long-term care systems. The objective of this study was to gain insights into the organizational and financial challenges affecting care transitions in long-term care systems in Germany, the Netherlands and Poland, and to inform potential system adaptations for improving care transitions in those countries.

**Methods:**

We employed an exploratory qualitative research design. In total, 22 semi-structured interviews involving 23 participants were conducted with providers representing primary care, hospitals, long-term care, and insurers/payers. Data were analyzed using qualitative content analysis with the use of programme ATLAS.ti. A deductive-inductive approach was applied to code the interviews.

**Results:**

Care transitions of older adults in long-term care systems in Germany, the Netherlands and Poland are suboptimal. Organizational challenges - such as communication, transfer of information, and coordination of resources have an immense impact on care transitions and require urgent attention. Among financial challenges, reimbursement plays a particularly crucial role in shaping care transitions across the three countries. Additionally, legal regulations, policies, the availability of protocols, and agreements between the providers might either facilitate or hinder care transitions.

**Conclusions:**

Our study adds to existing frameworks in this area by highlighting the importance of regulatory aspects and the availability of protocols for optimizing care transitions. These factors have received little attention in previous studies but can hamper care transitions if not adequately designed.

**Supplementary Information:**

The online version contains supplementary material available at 10.1186/s12877-025-06842-4.

## Key points

• At present, care transitions of older adults in long-term care systems in Germany, the Netherlands and Poland are not optimal.

• Organizational challenges such as communication, transfer of information, and coordination of resources have an immense impact on care transitions and need urgent attention.

• Among financial challenges, reimbursement plays a crucial role when it comes to care transitions in three countries.

**Supplementary Information:**

The online version contains supplementary material available at 10.1186/s12877-025-06842-4.

## Background

Older adults with complex health and social care needs might require care from different professionals in more than one setting and may therefore experience frequent care transitions [1, 2]. Care transition can be defined as “patient transfer between different locations or different levels of care within the same location” [3] p.556. These transitions represent vulnerable exchange points and should be optimized or avoided when possible [4]. Suboptimal care transitions have been linked to adverse events, hospital readmissions, low satisfaction with care, and high healthcare costs [4–9]. Furthermore, they are also associated with increased mortality and longer lengths of stay [9]. Poor-quality transitions are common [10]. Kapoor et al. [11] found that in nearly 4 of 10 discharges from the hospital back to long-term care (LTC) facilities, patients experienced adverse events, the majority of which were avoidable. Similar frequency of adverse events has been identified among community-dwelling older adults admitted to a skilled nursing facility [12]. Delayed discharges are another cause of concern. A systematic review by Landeiro and colleagues found that delayed hospital discharges among older adults are prevalent in most countries and are associated with high costs [13].

According to the World Health Organization, improving the safety of care transitions is a complex task and requires a range of strategies. These strategies should address the macro (health care system, LTC system), meso (health service organization) and micro (service delivery) levels [14]. Nonetheless, to appropriately tailor and implement strategies at different levels, understanding the context and the specific reasons for fragmented care transitions is essential [14, 15].

A recent systematic literature review by Wieczorek and colleagues suggests that broader organizational and financial aspects affect care transitions in LTC systems [16, 17]. Nevertheless, little is known about what stakeholders consider to be important barriers or facilitators of care transitions between the settings. Previous studies focused mainly on one type of transition [18–22] or discussed barriers and facilitators to the implementation of specific transitional care services instead [15, 22]. A wider system-level approach might prove more effective and sustainable than focusing on a single provider [23]. Moreover, compiling the perspectives of providers from various settings and insurers/payers might shed light on underlying key issues within LTC systems. Thus, a deep understanding of the role of organizational and financial factors in care transitions within LTC systems is an important starting point.

To address this research gap, a qualitative study was conducted with the objective of gaining insights into organizational and financial challenges that affect care transitions in LTC systems in Germany, the Netherlands and Poland. These countries were selected to represent different LTC system typologies. More information about each system and the population characteristics in each country can be found in Table 1.

**Table 1:** Comparison of population and LTC systems characteristics in Germany, the Netherlands and Poland

		Germany	The Netherlands	Poland
Population characteristics				
Life expectancy at age 65 (2023), in years		Male 17.9 Female 21.2	Male 18.9 Female 21.0	Male 16.2 Female 20.4
Share of the population aged 65 or over (2024)		22.4%	20.5%	20.5%
Share of the population aged 80 and over (2024)		7.2%	5.0%	4.4%
Limitations in daily activities in adults aged 65 and over (2024)	Some limitations	37.4%	42.7%	31.4%
	Severe limitations	18.2%	7.2%	14.6%
LTC system characteristics				
Typology		Private supply system	Need-based supply system	Residual public system
Characteristics of LTC type		Medium to high levels of supply in terms of beds and residents in institutions. Low shares of public expenditure. Low access restrictions – no means-testing and limited choice restrictions. Private expenditure (voluntary and out-of-pocket) is high.	Public expenditure level is average. High levels of supply in terms of beds and residents in institutions. Access is restricted by a high level of means-testing. Choice restrictions are low. Private expenditure (voluntary and out-of-pocket) medium.	Low levels of supply, low overall expenditure, beds, and residents in institutions. Access barriers seem low – no means-testing and low level of choice restrictions The share of public LTC expenditure is high. Private expenditure (voluntary and out-of-pocket) is low.
Total LTC spending as a share of GDP (2021)		2.5%^1^	4.4%	0.5%^1^
Formal LTC workers per 100 inhabitants aged 65 and over (2023)		5.5	8.3^E^	< 1
Beds in nursing and other residential long-term care facilities per 100 000 inhabitants (2023)		1187.3	1400.2	221.6
Percentage of care recipients aged 65 and over receiving care at home (2023)		83.3%	64.7%	Data not available

^1^ - Countries not reporting spending for LTC (social)

^2^ – Number of LTC beds in hospitals are not available in these countries

^E^ – estimated value

^*^2022

Source: Author’s compilation based on The Organization for Economic Cooperation and Development [24–27]

We used qualitative data collected from key informants in Germany, the Netherlands and Poland. This study plays a crucial role in identifying challenges and guiding potential system adaptations to optimize care transitions within LTC systems across the three countries.

## Methods

### Study design

The study was conducted in Germany, the Netherlands and Poland. We used an exploratory qualitative research design to understand the organizational and financial challenges in care transition in the LTC systems of the selected countries. We use COnsolidated criteria for REporting Qualitative research (COREQ) checklist [28] to report on the methods and results of our qualitative study (see Appendix 1).

### Participants and setting

We used a purposive sampling method to identify country key informants in LTC and care transition in Germany, the Netherlands, and Poland. To be included in the study, participants had to: (1) represent a provider of primary, hospital, or LTC, or insurers/payers; (2) have experience with care transitions of older adults; (3) be familiar with the LTC systems in Germany or the Netherlands, or Poland; (4) speak English, German or Polish.

We contacted 23 potential participants by e-mail (7–8 per country), and only one of the approached participants did not respond to the invitation to participate in the study. The invitation included information about the research team and the study, namely its aim, structure, duration, and possible outcomes. The time and the place of the interview were agreed on by all the participants. All the participants provided informed consent and voluntarily participated in the study.

Throughout our study, one of the main objectives was to provide the participants with respect, sensitivity, reduced risk of harm and exploitation. For this reason, we closely followed the principles outlined in the declaration of Helsinki. Our study was approved by the Ethical Committee at the Jagiellonian University (Poland) (approval number 1072.6120.54.2021) and Maastricht University (The Netherlands) (approval number FHML-REC/2021/079). Ethical approval for this study was not needed in Germany, as confirmed by the Institute for Health Care and Nursing Studies at Martin-Luther University Halle-Wittenberg. Informed consent (written and/or oral) was obtained from all participants prior to the interview.

### Data collection

Semi-structured interviews were conducted between June 2021 and February 2022. Interviews with Polish participants were conducted in Polish, and interviews with Dutch participants were conducted in English by the main researcher (E.W.), a female PhD candidate. Interviews in German were conducted by another researcher (C.S.) who is a male and holds a professor chair and PhD degree in economics. The two researchers had no relationship previously established with the respondents. Prior to this study, both researchers followed a course on how to conduct qualitative studies and had some practical experience in conducting interviews. No interviewers outside the research team were involved.

The interview guide was developed specifically for this study by the researchers, based on the findings of a prior literature review [16, 17] (see Appendix 2 for full interview guide). The guide was drafted, discussed, modified, and accepted by the research team. The first three interviews were used as a pilot test and confirmed that the interview guide was clear to participants, and thus, no adjustments were needed.

The interviews were scheduled at the place and time suggested by the participant. Most of the interviews (18 out of 22) were conducted online due to the COVID-19 pandemic restrictions. Three interviews were face-to-face and carried out in the workplace of the participants, and one participant provided the answers through e-mail. All interviews were carried out once (without repeated interviews) with only the participant(s) and an interviewer/s being present. Nonetheless, we also carried out one dyadic interview with two participants from the Netherlands. Thematic saturation marked the point at which data collection concluded i.e. information discovered in final interviews per country did not lead to new codes or significant changes in the results.

Each interview was audio recorded. We then transcribed the recordings using the Verbatim method and sent the transcripts to the participants for a member check. Only 2 participants provided some minor changes to the transcripts.

### Data analysis

All the interview data were coded and analyzed using the method of qualitative content analysis (software package ATLAS.ti Version 22). A deductive-inductive approach was used, i.e., the initial set of codes (themes/categories) was informed by a prior literature review [16, 17], while additional sub-codes (sub-themes/sub-categories) emerged from the interviews. Interviews in English and Polish with Dutch and Polish participants, respectively, were coded by the main researcher (E.W.) who is a native Polish speaker and fluent English speaker. Interviews in German were coded by another researcher (C.S.), who is a native German speaker, fluent Polish, and English speaker. The main researcher (E.W.) was also involved to ensure uniformity of coded data.

## Results

In total, 22 semi-structured interviews were conducted with 23 key country informants (8 from Germany, 8 from the Netherlands, one dyadic interview, and 7 from Poland). More information on participants’ characteristics is presented in Table 2. Each recorded interview lasted, on average, 52 min (range: 27–107 min).

**Table 2:** Participants’ characteristics

Interview Count	Participant Count	ID	Work Setting	Role	Participant code + characteristics
Germany					
1	1	P01	Hospital	Physician	P01, Hospital, Physician, Germany
2	2	P02	Hospital	Nurse	P02, Hospital, Nurse, Germany
3	3	P03	Primary care	Nurse	P03, Primary care, Nurse, Germany
4	4	P04	Long-term care	Nurse	P04, Long-term care, Nurse, Germany
5	5	P05	Long-term care	Nurse	P05, Long-term care, Nurse, Germany
6	6	P06	Long-term care	Nurse	P06, Long-term care, Nurse, Germany
7	7	P07	Payer/Insurer	Management	P07, Payer/Insurer, Management, Germany
8	8	P08	Payer/Insurer	Management	P08, Payer/Insurer, Management, Germany
The Netherlands					
9	9	P09	Hospital	Nurse	P09, Hospital, Nurse, The Netherlands
10	10	P10	Hospital	Nurse	P10, Hospital, Nurse, The Netherlands
11	11	P11	Primary care	Management	P11, Primary care, Management, The Netherlands
12	12	P12	Long-term care	Management	P12, Long-term care, Management, The Netherlands
13	13–14	P13,14	Long-term care	Nurse	P13,14, Long-term care, Nurse, The Netherlands
14	15	P15	Long-term care	Physician	P15, Long-term care, Physcian, The Netherlands
15	16	P16	Payer/Insurer	Management	P16, Payer/Insurer, Management, The Netherlands
Poland					
16	17	P17	Hospital	Social care worker	P17, Hospital, Social care worker, Poland
17	18	P18	Hospital	Nurse	P18, Hospital, Nurse, Poland
18	19	P19	Primary care	Nurse	P19, Primary care, Nurse, Poland
19	20	P20	Primary care	Physician	P20, Primary care, Physician, Poland
20	21	P21	Long-term care	Management	P21, Long-term care, Management, Poland
21	22	P22	Long-term care	Management	P22, Long-term care, Management, Poland
22	23	P23	Payer/Insurer	Management	P23, Payer/Insurer, Management, Poland

### Key findings

Following the qualitative content analysis methods described above, we first analyzed the data for each country separately, see Appendix 3, 4 and 5, and then, we prepared a comparative table, see Appendix 6. Below, we describe the key comparative outcomes per sub-theme and illustrate them with quotes.

#### Organizational challenges

**Fig. 1:**
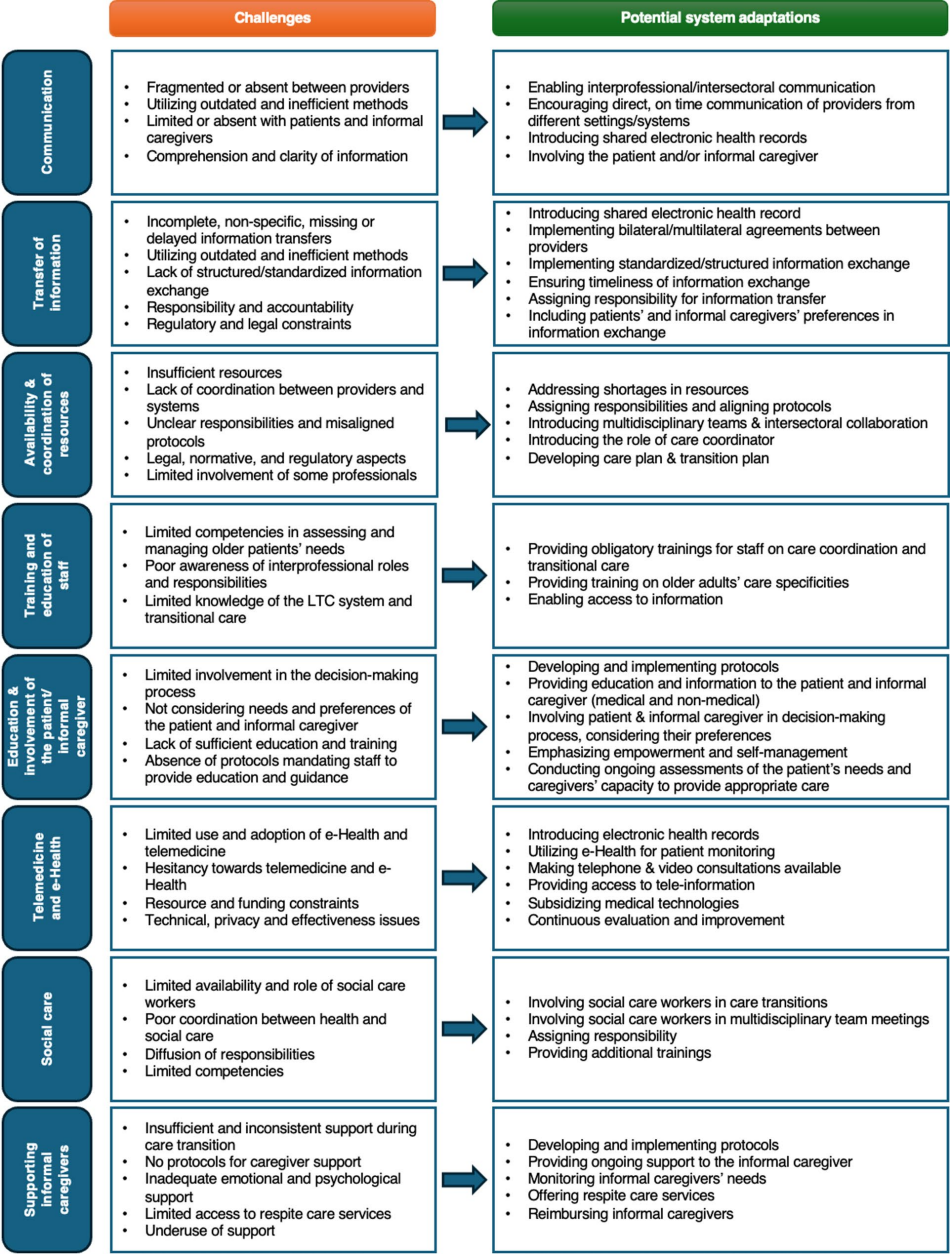
Organizational challenges in care transitions and potential system adaptations (summary based on the results from the three countries)

Figure 1 presents challenges associated with each organizational aspect identified in the interviews. It also outlines potential system adaptations proposed during the interviews with country informants from Germany, the Netherlands, and Poland.

### Sub-theme 1: communication

Key country informants in Germany, the Netherlands and particularly in Poland regarded communication as one of the most important organizational aspects that affect care transitions and indicated that communication is currently suboptimal and malfunctioning. A few informants from Germany and Poland, and more than half from the Netherlands, acknowledged the importance of effective interprofessional/intersectoral communication for care transitions.

“*But you have some communication issues between the care providers from the hospital to home. So how can you provide the information from the hospital to home timely*,* complete?*” *(*P10, Hospital, Nurse, The Netherlands)‬‬

“*There should be communication with the entity (organization)[…] and the patient should immediately go to such a place*,* and this is not the case.” (*P21, Long-term care, Management, Poland)

All informants from Poland were critical of the limited or absent communication between providers/sectors. A few German and Dutch informants argued that there are still some parts of the communication process that do not work well and should be addressed, but they noted that interprofessional/intersectoral meetings have a positive impact on communication. It was not stated whether such meetings take place within the Polish system.

Informants from the three countries argued about the importance of communication between providers regarding patients’ needs, as well as communication with the patients and their informal caregivers. A few participants stated that communication with informal caregivers in Germany and Poland is limited. In contrast, some Dutch informants suggested that social care workers, transfer nurses and community nurses play an important role in communicating with informal caregivers in the Netherlands. One informant from each country suggested that using electronic or digital methods, including e-Health, might be useful for communicating with providers/patients/informal caregivers.

### Sub-theme 2: transfer of information

Nearly all participants agreed that good transfer of information is essential for optimal care transitions, and there is still room for improvement. At present, transferred information is often incomplete, delayed, or missing, as stated by some informants in each country. Informants from each country suggested that standardized protocols for information exchange might facilitate this process. German and Dutch informants, along with one Polish informant, agreed that there is a need for electronic health records accessible to all providers/institutions involved in the care process. Some participants from Germany and one from Poland argued that outdated methods currently used in their countries (paper or fax) might have a negative impact on care transitions. According to a few German and Dutch informants, factors such as data protection laws might also restrict information transfer.

*“Sometimes it’s just the the laws. So the the documents that we have*,* we’re not allowed to transform from one institution to another.” (*P16, Payer/Insurer, Management, The Netherlands)

One participant from Germany and one from the Netherlands suggested that it is important to include patients’ preferences in the transferred information. A few Dutch informants were the only ones who argued that bilateral agreements between providers/institutions might improve the transfer of information. Half of the Polish informants argued that, in some cases, patients in the LTC system are responsible for carrying and transferring information to the next provider, which leads to suboptimal information exchange.

*“He (patient/caregiver) gets an information card in the corridor and information that tomorrow at 3 pm we will discharge the patient and that’s it.” (*P20, Long-term care, Management, Poland*)*

### Sub-theme 3: availability and coordination of resources

The availability of resources and their coordination might have an immense impact not only on the quality of care transitions but also on the direction of these transitions, meaning that some patients might not be transferred to the place where the provision of care would be most optimal (for instance, a transition from home to a nursing home), as suggested by the participants. Availability of resources was a challenge across all countries; however, it was particularly pronounced in Poland. Informants from each country acknowledged the important role of LTC infrastructure and availability of staff.

*“It’s difficult to have enough staff within the community care services at the moment […] then you can’t transfer patients either from the nursing home back to home*,* where they should have three times a day a visit from a nurse or from the hospital directly […] It ‘s the whole circle*,* it’s the chain of care where if there is something limited in one of them*,* then it is stuck. ” (*P09, Hospital, Nurse, The Netherlands)‬‬

More than half of Polish participants expressed their frustration regarding the inadequate LTC infrastructure and associated with it long waiting times to access LTC.

*“For the patient to get to this long-term care (in-home long-term care provided by a nurse)*,* there is a waiting list*,* and this waiting list is sometimes several years*,* several months.” (*P19, Primary care, Nurse, Poland*)*

According to Dutch informants, waiting time to access LTC institutions is a significant problem. In response to the growing care needs and limited LTC staff, two participants from Poland and one from Germany argued for the role of care assistants and the need for their involvement. Two Dutch participants suggested that strict criteria for accessing professional LTC might restrict some individuals from receiving help before it is “too late”.

Participants from all countries also acknowledged the need for comprehensive patient assessments. Some German informants, a few Dutch and more than half of Polish participants agreed that to deliver safe and seamless care transitions for older adults, a care coordinator is needed. In Germany and the Netherlands, some professionals in certain settings are already fulfilling that role (e.g. transfer nurses in the hospitals in the Netherlands). However, a care coordinator is not present anywhere in the Polish LTC system. Additionally, informants argued for the need for better interprofessional and intersectoral collaboration. Some participants highlighted the important role of clearly defined professional responsibilities, arguing that a lack of clarity may lead to a risk-averse behavior among the staff and even to the transfer of patients to hospitals.

*“…the responsibilities of the professions must be clear…if this is not clarified as quite frequently in Germany*,* then you have the phenomenon that everyone wants to be on the safe side. And then that means for the nurses they rather have the residents transferred to the hospital rather than have a look at them…” (P02*,* Hospital*,* Nurse*,* Germany)*

Moreover, a few informants from Germany and the Netherlands suggested that multidisciplinary team meetings might improve collaboration.

*“So if it’s routine care*,* having this interdisciplinary multidisciplinary collaboration […] and so the routine of doing so is very important.*” *(*P09, Hospital, Nurse, The Netherlands)

They also argued about the important role of support from the management, implementation of transitional care, and the need for care/transition planning.

### Sub-theme 4: training and education of staff

There was consensus among German, Dutch, and Polish key informants that staff involved in the care process should be well-trained and educated to deliver optimal care transitions. Two participants from Poland and one from the Netherlands argued that, at present, staff members’ knowledge regarding the work of other professionals/settings involved in transitional care is limited, which negatively impacts care quality.

*“And my experience is that many caregivers*,* professionals don’t even know what the other profession does or what’s available.” (*P09, Hospital, Nurse, The Netherlands*)*

More than half of informants from Poland, a few from the Netherlands and one from Germany underlined the need to provide the staff with education regarding transitional care as well as communication and transfer of information. Dutch and Polish participants even argued that such education should be an essential part of each training program. Currently, no training exists for staff regarding transitional care, as stated by the German and Polish informants.

According to two informants from the Netherlands and one from Poland, the effectiveness of education and training should be improved. One German and two Polish participants also argued about the importance and availability of training for care assistants. In their view, provision of such training could improve care transitions of older adults by increasing the availability of LTC staff.

*“Now there is a fashion for care assistants*,* so there are special trainings.”* (P17, Hospital, Social care worker, Poland)

### Sub-theme 5: education and involvement of the patient and/or informal caregiver

Country informants agreed on the importance of well-educated and informed patients and informal caregivers and on the need for their stronger involvement in the care process. At least half of the informants in the three countries argued for the importance of providing multidimensional education and information to the patient and/or informal caregiver, and the positive impact it might have on care transitions.

*“[…] because if the client or the family are rightly informed*,* they know what is going to happen and they know what they need to do.” (*P13,14, Long-term care, Nurse, The Netherlands)

A few Polish participants stated that patients/informal caregivers often lack essential knowledge and are not prepared for care transitions. Participants from three countries agreed that it is vital to involve informal caregivers in the care process. In their view, at present, informal caregivers are not involved enough in the care process. Half of the informants from Germany and the Netherlands discussed the importance of considering patients’ and caregivers’ needs and preferences. According to half of German participants, these preferences are not always known, which contributes to suboptimal care transitions.

*“[…] because the patients needs are not considered. So there are other mechanisms behind the decisions*,* whether patient is transitioned or not.” (*P03, Primary care, Nurse, Germany*)*

Furthermore, some German and one Dutch informants suggested the need for the involvement of the patients and informal caregivers in the decision-making process, while half of German participants declared that this kind of involvement is limited at present.

### Sub-theme 6: telemedicine and e-Health

According to country informants, the use of telemedicine and e-Health in transitional care could be improved in each country, and particularly in Germany. At present, none of the countries possess electronic health record that is widely used by all providers. According to nearly all German and a few Dutch and Polish informants, the use of telemedicine and e-Health is limited in their countries.

*“We are still in the really beginning in Germany with implementation of e-Health.” (P03*,* Primary care*,* Nurse*,* Germany)*

Nevertheless, informants agreed on the importance of electronic devices to monitor patients at home. A few German and nearly all Polish participants pointed out the importance of video consultations and tele-nursing services. Informants argued for the need for digitalization in LTC and the introduction of electronic health records, where information about the patients could be easily accessible to those involved in the care process.

*“If we can have electronic files about the patient*,* it does help because then it’s easier for different caregivers to find out about the elderly and what its ailments are.” (P16*,* Payer/insurer*,* Management*,* The Netherlands)*

*“If you talk about video consultations […] this is a very good option to keep them at home*,* to keep them away from the risks that they have in hospital.” (*P02, Hospital, Nurse, Germany*)*

On the other hand, two participants from Germany, one from the Netherlands and one from Poland suggested that the complexity of older adults’ needs might limit the use or reduce the effectiveness of telemedicine and e-Health.

### Sub-theme 7: social care

Country informants claimed that the impact of social care on care transitions is ambiguous. Social care plays a vital role, but the involvement of social care workers in transitional care is limited. Informants from the three countries agreed that social care workers could help by preparing care transitions, e.g. in hospitals. Half of Dutch informants and some Polish informants also agreed that there is a need for social care workers to provide comprehensive care.

*“Well*,* in our organization*,* they (social care workers) are essential. They have a major role.” (*P15, Long-term care, Physician, The Netherlands)

On the other hand, a few participants from Germany and the Netherlands had mixed feelings regarding the involvement of social care workers in discharge planning. From the perspective of German informants, giving this role to social care workers might lead to a diffusion of responsibility. Dutch participants argued that the discharge role could be better performed by nursing staff.

However, it is important to involve social care workers in interprofessional/intersectoral meetings, as suggested by German participants. Moreover, according to the German informants, social care institutions could support the patient/informal caregiver to cover their LTC costs, which might have an impact on the direction of the care transitions.

*“The social welfare office always steps in when the patient can no longer afford to pay (for LTC).” (* P08, Payer/Insurer, Management, Germany)

Nevertheless, communication with social care institutions is not always optimal, as suggested by the German and Polish participants. Also, informants from Poland complained about the insufficient number of social care workers, which negatively affects care transitions.

### Sub-theme 8: supporting informal caregivers

Currently, the three countries do not address informal caregivers sufficiently due to restricted access to information and education/training, and limited involvement. For instance, in the Polish LTC system, informal caregivers are left on their own and need to search for support and information themselves. According to a few Polish informants, this might be the result of the absence of a formal requirement to provide support to informal caregivers. Informants from all countries agreed on the important role of education and training for informal caregivers, and the positive impact it might have on the care transitions of older adults. One informant from Germany and two from the Netherlands indicated that psychological and social needs of informal caregivers should be addressed by providing information and support. Some German and one Polish participant argued about the crucial role of respite care services, which provide temporary relief to informal caregivers.

*“[…] but also because of the risk of caregiver burden. I think it’s also important that the community nurse*,* for example […] offer some psychological or social support.” (*P10, Hospital, Nurse, The Netherlands)

#### Financial challenges

### Sub-theme 9: reimbursement

Participants suggested a sufficient level of LTC financing would help to optimize care transitions are in their countries, because it would affect not only the availability of facilities but also of staff. As a result, more than half of the Polish and a few Dutch informants argued for higher or better-estimated reimbursements of LTC facilities and competitive or higher salaries for LTC staff. One German and some Dutch informants added that the current reimbursement of some providers in their countries did not correspond to the needed workload or care provided. Participants from each country suggested that the lack of reimbursement for interprofessional and intersectoral collaboration might also negatively impact care transitions. Moreover, German and Polish informants spoke in favor of reimbursing telemedicine and training for informal caregivers, while one Dutch participant considered it vital to include physiotherapy in reimbursements.

*“And from the physical therapist what I told you this is not in the regular insurance. So patients have to pay more to have physical therapists in their insurance.” (*P10, Hospital, Nurse, The Netherlands)

Half of informants from Germany and one from the Netherlands and one from Poland suggested that organizations/institutions should step in and provide support in case of unaffordable LTC. Nonetheless, in their view, there might be a reluctance among organizations/institutions to take this role.

A few German, Dutch and Polish participants argued that activity-based payments for providers might have a negative impact on care transitions. The effectiveness of value-based payments was also questioned by the participants. Some Polish participants were the only ones to argue about the important role of charities, NGOs and volunteers in providing financial support.

Participants from three countries agreed that particularly out-of-pocket payments might affect the direction of care transitions. In their view, older adults and their caregivers might have restricted access to formal LTC due to their inability or unwillingness to cover high out-of-pocket costs.

*“[…] if it is financing the so-called commercial*,* then the patient simply cannot afford it and stays at home.” (*P19, Primary care, Nurse, Poland)

*“Well*,* I think there might be situations where the the amount that patients have to pay…for care can be a problem.” (*P15, Long-term care, Physician, The Netherlands)

### Sub-theme 10: rewards

The topic of rewards in LTC has divided the participants. Some Dutch and German informants had mixed or even negative opinions regarding the use of financial rewards, while Polish participants were more positive and were even in favor of their implementation. Informants in all countries realized the potential of financial rewards to improve quality of care and, ultimately, care transitions while at the same time acknowledging their limitations. Some informants from Germany and one from Poland suggested that it might be problematic to identify the party responsible for the improving quality of care transitions. Currently, the system of financial rewards is not present within the Polish and German systems, as stated by the participants, but two Dutch informants declared that such a system was already introduced in the Netherlands. One German and a few Dutch participants questioned the effectiveness of these rewards in the long term. Half of informants indicated that the internal motivation of staff to provide good care should be prioritized over financial rewards.

### Sub-theme 11: penalties

Country informants had rather mixed feelings or were even hesitant about the use of financial penalties in their LTC systems. Some participants from Germany and Poland had limited knowledge about the financial penalties and the impact they might have on care transitions. Nevertheless, informants from the three countries suggested that financial penalties could be issued for inappropriate care, adverse events, and various types of abuse. The system of penalties is already implemented in the Netherlands and Poland, as stated by participants. One Dutch and several Polish informants argued that financial penalties could further burden already strained LTC budgets. Informants from all three countries agreed that measuring the quality of care and identifying a responsible party might be challenging.

*“How can you really prove that a hospital released the patient too early? I imagine that’s very difficult.” *(P04, Long-term care, Nurse, Germany)

One participant from the Netherlands argued that the effect of financial rewards is short-lived.

*“It does help*,* but it only helps for a very short period of time and an incentive is just a year or two years*,* whatever*,* and then you get used to it and it doesn’t work anymore.” (*P16, Payer/Insurer, Management, The Netherlands*)*

## Discussion

This study aimed to explore organizational and financial challenges in care transitions in LTC systems in Germany, the Netherlands and Poland based on country informants’ opinions. The objective of the study was to inform improvements in care transitions in LTC systems in these countries. Nevertheless, the findings may also apply to other contexts and countries facing challenges in their long-term care systems.

Our results demonstrated that the Polish LTC system is ill-equipped to deliver optimal care transitions. First and foremost, there is a need to address the LTC infrastructure, which remains insufficient in terms of staff, facilities, and beds. A report by OECD [24] indicated that Poland has one of the lowest numbers of LTC workers and LTC beds among OECD member countries. Even though the majority of LTC in Poland is provided by informal caregivers, the current infrastructure and staffing levels are inadequate to address the LTC needs of older persons [29–31]. As our findings suggest, the focus should be on ensuring satisfactory reimbursement for LTC providers and reducing out-of-pocket payments. According to Błędowski [32], increasing salaries for LTC staff in Poland is one of the necessary steps to expand the LTC workforce. Moreover, unaffordable out-of-pocket payments require particular attention, as they influence the direction of care transitions in Poland and may lead to unmet LTC needs [24, 33, 34]. Additionally, our results showed an urgent need to improve coordination, communication, and transfer of information among those involved in care transitions, which remain key weaknesses of the Polish LTC system. The 2020 report of the Patient Ombudsman [35] revealed that the patient’s right to medical records and health information was insufficiently respected in Poland. Nonetheless, in recent years, some progress has been made toward improving care coordination within the Polish healthcare system. Since October 2022, a new role – the care coordinator – has been introduced, operating within family physician practices. However, their coordinating function is currently limited to primary and outpatient specialist services [36].

Based on our findings, the German LTC system is also insufficiently prepared to deliver optimal care transitions due to a shortage of staff needed to meet current LTC demands. A study by Gruber et al. [37] found that staffing shortages in home care are present throughout the country. Moreover, our study revealed that patients and their informal caregivers are not adequately involved in the care process, and communication with them remains limited. This is corroborated by a 2017 report suggesting that the patient and informal caregiver are often inadequately involved in the decision-making process [38]. Additionally, communication problems between patients and healthcare professionals have also been reported [39]. Furthermore, we found deficiencies in the transfer of information within the German LTC system. Möller and Makoski [40] argued that although letters are mandatory in Germany, the transferred information is often incomplete or delayed.

Our study results demonstrated that the Dutch LTC system is also not yet fully prepared to deliver optimal care transitions for older patients. This is an important finding, given the relatively high LTC expenditure in the Netherlands (4.1% of GDP in 2019) and the high availability of staff compared to other OECD member countries (around 8 LTC workers per 100 people aged 65 and over) [24]. A recent study by Groenvynck et al. [41] carried out in the Netherlands also found that informal caregivers often perceive care transitions as suboptimal and fragmented. We identified communication problems as well, particularly concerning the transfer of information between providers and overall communication among stakeholders. Daliri et al. [42] also reported that insufficient information transfer is one of the leading causes of medication-related problems following discharge in the Netherlands. Similarly, Poldervaart et al. [43] argued that some proportion of transition safety incidents in the Dutch LTC system could be attributable to poor information transfer or inadequate communication.

The results of our study demonstrated some degree of similarity across the three countries regarding problems with communication and the transfer of information, which have a significant impact on care transitions, as widely discussed in the literature [44, 45]. A potential solution to this problem could be the implementation of electronic health records, as also indicated by country informants in our study. However, the informants also acknowledged privacy issues that could limit their adoption. Similarly, Keshta & Odeh [46] argued that electronic health records enable easier data exchange while pinpointing important security and privacy concerns. Pohlmann et al. [47] indicated multiple barriers to implementing electronic health records, such as documentation standards, interoperability, and political structure. As illustrated by the example of electronic health records, the implementation of system adaptations requires understanding not only of the LTC system itself but also of the broader organizational and regulatory environment.

We also found some key differences between the factors affecting care transitions in the three countries, primarily due to variations in the provision and financing of care. According to Ariaans, Linden & Wendt [27], the LTC systems of the countries included in this study represent different typologies: Germany is considered as a private supply system, Poland as a residual public system, and the Netherlands as a need-based supply system.

According to the model of care provision aspects, organizational and financial aspects might impact the care transitions of older adults [16]. Other factors, such as contextual, political, cultural, social, historical, and economic factors, also influence LTC systems [48]. Our findings add to that body of knowledge by pointing out that legal regulations, policies, and the availability of protocols and agreements between providers may also either promote or hinder care transitions. For instance, policies and guidelines could also be potential risk factors for avoidable transitions due to restrictive data protection measures, which might hamper communication between providers [39].

Given the complexity and interplay between the organizational and financial aspects that can potentially affect care transitions, improving the functioning of the LTC system requires comprehensive and multidimensional strategies. Focusing solely on one component, for instance, introducing a care coordinator or electronic health records, is not sufficient. It is important to remember that even the best strategies will not yield the expected results if governance, organizational values, legal regulations, protocols and policies are not aligned with the principles of integrated care. As emphasized in the Handbook of *Integrated Care* [49], integrated care should be viewed not as a collection of specific interventions, but as an overarching approach that influences how we design, manage and coordinate the services for those with complex care needs. Moving away from conventional health care to integrated care requires appropriate governance and accountability [49]. As identified in this study, developing and implementing protocols, understood as formal set of rules and procedures across care settings, is an important factor that could potentially lead to the optimization of care transitions. Moreover, providers should be equipped with tools that enable them to implement new ways of working while minimizing additional burdens on already strained resources.

Similar importance is also attributed to culture and values, both organizational and personal. Culture and values might affect how people perform certain tasks, what kind of choices they make and the priorities they set. Therefore, it is crucial to take these multi-layered and multidimensional factors into account when aiming to achieve effective change within the organizations [50].

Optimizing care transitions should be a priority in every LTC system worldwide, as it can bring measurable benefits not only to patients and communities but also to healthcare professionals, and the healthcare system as a whole [51]. For instance, transitional care services for older adults proved to reduce mortality, readmission and readmission days [52]. Additionally, implementing coordinated care in primary care may also improve healthcare professionals’ job satisfaction and reduce unnecessary workload. However, the impact on care providers is not uniform and depends on the type of integration and the broader context. One the other hand, more studies are needed to fully understand the effects of optimizing care transitions on healthcare staff and to explore the impact of systemic changes.

### Strengths and limitations

With our qualitative study, we were able to capture rich data on a previously under-researched area. Moreover, this study builds on the model of care provision aspects that affect care transitions and adds new components, as discussed above. However, we recognize that our study has certain limitations. One major limitation is that the key informant group included only providers and payers/insurers. It is of great importance to involve the perspectives and opinions of patients as well as those of their caregivers, not only in future research but also in future policymaking. Thus, it would be important to investigate their opinions and expand the study to additional countries for a broader comparison. Another limitation is the relatively small number of informants per country. Nevertheless, the sample size allowed us to reach thematic saturation during the data collection. Nevertheless, given the complexity of the long-term care systems, we encourage researchers to increase their sample sizes and diversify the participants to improve the generalizability of findings.

## Conclusion

Our findings indicate that care transitions for older adults in long-term care systems in Germany, the Netherlands, and Poland remain suboptimal. Considerable improvements are necessary to support safe and coordinated transitions across settings. Organizational challenges related to communication, information exchange, and coordination of resources have been identified as critically important factors in care transitions and warrant immediate attention. Additionally, financial challenges, namely reimbursement, play an important role, although their adequacy varies across the three countries. Our study adds to the previous frameworks in this area by highlighting the importance of regulatory aspects and protocols for optimizing care transitions, which have not been the focus of previous studies but can hinder care transitions if not adequately designed.

## Supplementary Information

Supplementary Material 1.

## Data Availability

The datasets used and/or analyzed during the current study are available from the corresponding author on reasonable request.
