# Supplementary material for: Organizational and financial challenges in care transitions: a qualitative study of long-term care systems in Germany, the Netherlands and Poland
Source: BMC Geriatr. 2025 Dec 11;26:65. doi: 10.1186/s12877-025-06842-4 (PMC12817419; doi:10.1186/s12877-025-06842-4)
Supplement: Supplementary file 1 — Supplementary Material 1. [file 12877_2025_6842_MOESM1_ESM.docx]

**Organizational and financial challenges in care transitions: A qualitative study of long- term care systems in Germany, the Netherlands and Poland**

**Table of contents**

1. **Appendix 1. COREQ (COnsolidated criteria for REporting Qualitative research) Checklist**
2. **Appendix 2. Interview guide**
3. **Appendix 3. Key findings – Germany**
4. **Appendix 4. Key findings – The Netherlands**
5. **Appendix 5. Key findings – Poland**
6. **Appendix 6. Basic findings from the interviews**

**Appendix 1. COREQ (COnsolidated criteria for REporting Qualitative research) Checklist**

Developed from:

Tong A, Sainsbury P, Craig J. Consolidated criteria for reporting qualitative research (COREQ): a 32-item checklist for interviews and focus groups. International Journal for Quality in Health Care. 2007. Volume 19, Number 6: pp. 349 – 357

| **Item No** | | **Guide Questions/Description** | **Reported on Page #** |  |
| --- | --- | --- | --- | --- |
| **Domain 1: Research team and reflexivity** | | | |  |
| **Personal Characteristics** | | | |  |
| 1. Interviewer/ facilitator | | Which author/s conducted the interview or focus group? | Pg 8 |  |
| 2. Credentials | | What were the researcher’s credentials? E.g., PhD, MD | Pg 8 |  |
| 3. Occupation | | What was their occupation at the time of the study? | Pg 8 |  |
| 4. Gender | | Was the researcher male or female? | Pg 8 |  |
| 5. Experience and training | | What experience or training did the researcher have? | Pg 8 |  |
| **Relationship with participants** | | | |  |
| 6. Relationship established | | Was a relationship established prior to study commencement? | Pg 8 |  |
| 7. Participant knowledge of the interviewer | | What did the participants know about the researcher? e.g. personal goals, reasons for doing the research? | Pg 8 |  |
| 8. Interviewer characteristics | | What characteristics were reported about the interviewer/facilitator? e.g. Bias, assumptions, reasons and interests in the research topic | Pg 8 |  |
| **Domain 2: study design** | | |  |  |
| **Theoretical framework** | | |  |  |
| 9. Methodological orientation and Theory | What methodological orientation was stated to underpin the study? e.g. grounded theory, discourse analysis, ethnography, phenomenology, content analysis | Pg 7 |  |  |
| **Participant selection** | | |  |  |
| 10. Sampling | How were participants selected? e.g., purposive, convenience, consecutive, snowball | Pg 7 |  |  |
| 11. Method of approach | How were participants approached? e.g., face-to-face, telephone, mail, email | Pg 7 |  |  |
| 12. Sample size | How many participants were in the study? | Pg 10 |  |  |
| 13. Non-participation Setting | How many people refused to participate or dropped out? Reasons? | Pg 7 |  |  |
| 14. Setting of data collection | Where was the data collected? e.g., home, clinic, workplace | Pg 9 |  |  |
| 15. Presence of nonparticipants | Was anyone else present besides the participants and researchers? | Pg 9 |  |  |
| 16. Description of sample | What are the important characteristics of the sample? e.g. demographic data, date | N/A |  |  |
| **Data collection** | | |  | No |
| 17. Interview guide | Were questions, prompts, and guides provided by the authors? Was it pilot tested? | Pg 8 |  |  |
| 18. Repeat interviews | Were repeat interviews carried out? If yes, how many? | Pg 9 |  |  |
| 19. Audio/visual recording | Did the research use audio or visual recording to collect the data? | Pg 9 |  |  |
| 20. Field notes | Were field notes made during and/or after the interview or focus group? | N/A |  |  |
| 21. Duration | What was the duration of the interviews or focus group? | Pg 10 |  |  |
| 22. Data saturation | Was data saturation discussed? | Pg 9 |  |  |
| 23. Transcripts returned | Were transcripts returned to participants for comment and/or correction? | Pg 9 |  |  |
| **Domain 3: analysis and findings** | | |  |  |
| **Data analysis** | | |  |  |
| 24. Number of data coders | How many data coders coded the data? | Pg 9 |  |  |
| 25. Description of the coding tree | Did the authors provide a description of the coding tree? | Appendix |  |  |
| 26. Derivation of themes | Were themes identified in advance or derived from the data? | Pg 9 |  |  |
| 27. Software | What software, if applicable, was used to manage the data? | Pg 5 |  |  |
| 28. Participant checking | Did participants provide feedback on the findings? | N/A |  |  |
| **Reporting** | | |  |  |
| 29. Quotations presented | Were participant quotations presented to illustrate the themes/findings? Was each quotation identified? e.g., participant number | Pg 11-26 |  |  |
| 30. Data and findings consistent | Was there consistency between the data presented and the findings? | Pg 11-26 |  |  |
| 31. Clarity of major themes | Were major themes clearly presented in the findings? | Pg 11-26 |  |  |
| 32. Clarity of minor themes | Is there a description of diverse cases or a discussion of minor themes? | Pg 11-26 |  |  |

**Appendix 2. Interview guide**

**M/F**

**Provider / Payer, insurer / Policy-maker**

**Age:**

**Institution:**

**Country:**

**Experience with “care transitions”:**

**Qualitative interview introduction**

This interview will take approximately 45-60 minutes.

**Primary goal:** The primary goal of this interview is to identify organizational and financial aspects that affect care transition in the long-term care systems. We are interested in your experiences and opinions.

**Background Information**

This interview is performed as part of the European TRANS-SENIOR project. The dual focus of the TRANS-SENIOR research is on avoiding unnecessary care transitions and improving care for transitions that are needed. Understating organizational and financial factors that affect care transition is crucial to improve the quality of transitions and continuity of care.

Transitional care refers to a set of actions designed to ensure the coordination and continuity of health care as patients transfer between different locations or different levels of care within the same location”.

Optimizing care transitions means avoiding unnecessary care transitions and improving care for transitions that are needed.

**ORGANIZATIONAL ASPECTS**

Do you think that there is relation between how long-term care is organized and the direction of the transition?

- What kind of relation is there?
- Is there relation that older adults are more likely to be institutionalized?

Do you think that there is a relation between the **organizational aspects** and care transitions between the settings?

- Can you name any organizational aspects that have an impact on care transitions between the settings?
- In your experience does the communication among involved professional groups have an impact on care transitions between the settings?
- How?
- In your experience does the transfer of information and care responsibility of the patients have an impact on care transitions between the settings?
- How?
- In your experience does the coordination of resources (involving nurses, pharmacists, transition care manager or program) have an impact on care transitions between the settings?
- How?
- In your experience does the training and education of staff have an impact on care transitions between the settings?
- How?
- In your experience does the e-health have an impact on care transitions between the settings?
- How
- In your experience does the education and involvement of the patient and family have an impact on care transitions between the settings?
- How?
- In your experience does the social aspects (social care)(availability of social care worker) have an impact on care transitions between the settings?
- How?

Can you think of any other important aspects that affect care transfers?

Do you think that some of these organizational aspects are more important than others? Could you name any?

- Why do you think they are more important?

What is in your opinion optimal care transition? Do you think that care transition of older patients is optimal according to that definition?

**FINANCIAL ASPECTS**

Do you think that there is relation between how long-term care is financed and the direction of the transition?

- What kind of relation is there?
- Is there relation that older adults are more likely to be institutionalized?

Do you think that there is a relation between the **financial aspects** and care transitions between the settings?

- Do you think there is an impact of financial aspects on care transitions between the settings?
- Can you name any of the financial aspects or mechanisms that have an impact on care transition?

In your experience does the way providers are reimbursed have an impact on care transitions?

How?

- Do you think that activity-based payment methods could have an impact on care transitions?
- If yes, how?
- Do you think that value-based payments methods could have an impact on care transitions?
- If yes, how?

Do you think that pay-for-performance payment mechanism could have an impact on care transtions?

- - Do you think that rewarding for the role of care coordinator have an impact on care transitions?

How?

In your experience/opinion what is the relation between the availability of financial rewards and penalties and care transitions?

- - Can you say more about it?

Do you think that some of these financial aspects are more important than others? Could you name any?

- Why do you think they are more important?

In your opinion what should be done to improve care transition between the settings?

- Can you think of any solutions to optimize care transitions?

Is there anything more you would like to add?

**Appendix 3. Key findings – Germany**

Semi-structured interviews with 8 key country informants enabled us to identify important sub-themes for organizational and financial themes affecting care transitions of older adults in Germany. We interviewed participants representing following: providers - 1 from primary care, 2 from hospital, 3 from long-term care, and 2 insurers/payers.

Majority of the informants agreed that care transitions in the German long-term care system are not optimal, and still, a lot has to be done if Germany is to deliver safe and seamless care transitions for older adults. Participants argued that broader organizational and financial aspects might affect not only the quality-of-care transition but also the direction. Below we present different organizational and financial aspects that affect care transition in the German long-term care system.

**Organizational challenges**

Country informants in Germany agreed that organizational aspects and especially communication, transfer of information, coordination of resources, education and involvement of the patient and informal caregivers might have an immense impact on care transitions of older adults. In their view, there is a room for improvement regarding many organizational aspects.

**Sub-theme 1: Communication**

According to country informants, there is a strong need for good communication between different professionals and sectors involved in care process. Participants suggested that especially availability of regular round-table meetings with professionals from different settings and sectors might lead to optimization of care transitions. Moreover, they argued that knowing involved professionals and institutions personally might ease communication. Participants also suggested that there is a need for a central actor that would facilitate communication among involved groups about the availability of LTC facilities.

*‘I find it difficult that there are so many parallel structures…so we have a wide variety of providers in the region…so this parallel means that there is somehow no central path that you can take, but you can only try it in very, very many places’ (P01, Hospital, Physician, Germany)*

According to country informants, there is not only a need for personal communication with the patients and family, but also for the communication of 3 sides – the patient, sending and receiving setting. Participants declared that current communication with the patients and their caregivers is very limited. Furthermore, they agreed that there is a need for better communication between the settings about patients’ needs and planned transitions of vulnerable patients. Participants were in favor of on-time, verbal, detailed and electronic communication. On the other hand, using outdated methods (e.g., on paper) might be a barrier to good communication. One participant also suggested that the use of professional language/formal language that is incomprehensible for other groups might lead to miscommunication and lack of understanding among involved groups.

**Sub-theme 2: Transfer of information**

Majority of the country informants agreed that the current transfer of information between different professionals involved in care transitions is suboptimal and that there is room for improvement. According to half of the participants, transferred information is often non-specific, incomplete and/or delayed.

*‘…what mostly comes across is a piece of paper that says name…and that's usually it. Nothing is settled there. What is the family member's name? What does a man need? What does the person have? Everything comes days later. Always.’ (P04, Long-term care, Nurse, Germany)*

However, it also happens that the information is never delivered. As a result, informants suggested that the use of a standardized protocol for information exchange could improve the transfer of information between providers. On the other hand, they declared that even though some parts of Germany introduced standardized protocol, it is not used in routine care. Furthermore, participants argued that not only what kind of information is transferred is important, but also how the information is transferred. According to informants, outdated methods (on paper, fax) of information exchange should be replaced with electronic health information exchange, especially electronic patient records.

*‘But if you look at the documentation in nursing homes, but also in hospitals, it's still very much paper based. So which you would not expect in the 21st century, I guess, but it's mostly or very often in files.’ (P02, Hospital, Nurse, Germany)*

*‘Ideally, of course, you want to have it (information) in an electronic patient file.’ (P02, Hospital, Nurse, Germany)*

Another factor that might hamper information exchange among professionals and organizations refers to the data protection regulations in Germany.

*‘…it's all data protection issues, of course, I know that too.’ (P04, Long-term care, Nurse, Germany)*

**Sub-theme 3: Availability & coordination of resources**

More than half of German informants agreed that there is a need for good interprofessional and intersectoral collaboration among all involved in the care process. Nonetheless, first structures and routines for intersectoral and interprofessional collaboration need to be adapted, and there needs to be support from the management. Moreover, according to participants, professionals and organizations should have clearly defined responsibilities, meet on a regular basis and be aware of how other settings work. One participant suggested that unclear responsibilities of the professionals and limited use of case conferences often lead to suboptimal transitions in the German long-term care system.

*‘…the responsibilities of the professions must be clear…if this is not clarified as quite frequently in Germany, then you have the phenomenon that everyone wants to be on the safe side. And then that means for the nurses they rather have the residents transferred to the hospital rather than have a look at them…’ (P02, Hospital, Nurse, Germany)*

*‘…and of course, there should be things like case conferences, which we…know about, but which we don't do really.’ (P02, Hospital, Nurse, Germany)*

Informants also argued that the presence of case management or case manager could lead to optimization of care transitions. They suggested that case manager (e.g., advanced practice nurse) role should be to address social aspects and to prepare the care plan while optimizing care for the patient. They also argued that care planning, transition planning and carrying out patients’ needs assessment are crucial to ensure a smooth care transition process. Nevertheless, all these efforts would be for nothing if LTC infrastructure was incapable of addressing all the care needs. According to informants in Germany, limited availability of staff, especially in LTC and limited availability of places in LTC facilities might have an impact on care transitions. Furthermore, lack of coordination between health and long-term care providers, separate financing streams for health care and long-term care, certain normative and legal regulations, and organizational protocols that are inflexible to be adapted to the patient’s situation might also negatively affect care transitions. On the other hand, legal regulations might also have a positive effect on care transitions. This refers specifically to regulations regarding discharge planning and loosened regulations regarding hiring (health) care personnel from abroad. Besides that, participants suggested that there is a need for stronger involvement of primary care and care assistants during the transition process. Moreover, informants in Germany argued that insurance companies might also play an important role in planning and organizing care transitions.

*‘What I also find important is the role of the health insurance company… they (insurance company) just have someone on site for the region who knows the structures very well. Knows many facility managers personally and who plays a major role in many placements. So the insurance company is not always the opponent, but also often a player who really helps to find the right thing for the patient.’ (P01, Hospital, Physician, Germany)*

**Sub-theme 4: Training and education of staff**

According to participants, even though in some parts of the German system, professionals are well-trained and educated to provide transitional care, there is still room for improvement. Currently, to be a nurse, individuals need to follow comprehensive education programs that positively impact their competencies.

*‘So the training of the nursing staff, who learn for three years, is very extensive here, with a lot of theoretical hours, but also a lot of practical hours […] anyone who is now doing nursing training in Germany […] have to get to know the various areas (providers) during their training and also do practical training.’ (P07, Payer/Insurer, Management, Germany)*

Participants argued that the added value of the education program is that individuals get to know how different providers operate. Country informants argued that staff knowledge about how the care is organized in other settings is crucial for smooth care transitions. Additionally, half of participants agreed that professionals involved in older adults’ care transitions should be well-trained and have core competencies. For this reason, staff needs to follow mandatory training courses.

*‘We have mandatory training courses that we complete every year, some - every two years.’ (P05, Long-term care, Nurse, Germany)*

Besides that, some employers offer additional training to the staff. As a result, some staff is well trained to provide transitional care to older adults, according to some informants.

*‘The hospitals are very, very well set up in terms of education.’ (P06, Long-term care, Nurse, Germany)*

Nonetheless, few participants argued that still more attention should be paid to the training regarding communication/transfer of information. Moreover, informants proposed that there is a need for increasing awareness of the staff regarding transitional care, as currently there are no such training. Additionally, some participants stated that staff lack competencies to assess patients’ needs and to tailor care and sometimes is not trained enough to perform activities independently.

*‘I think it would be important for the medical assistants in the family doctor's practice to be much more involved, much more knowledge, maybe trained.’ (P05, Long-term care, Nurse, Germany)*

At last, availability of one-year training for care assistants providing all non-medical services was seen by one informant as a facilitator that could improve transitional care of older adults by increasing the availability of LTC staff.

**Sub-theme 5: Education and involvement of the patient and/or caregiver**

Half of informants agreed on the importance of providing information and education to the patient and the caregiver. Few participants stated that such education and information are already provided in the German long-term care system.

*‘We have had training courses for relatives for many years, especially in the field of dementia. I regularly do courses there twice a year.’ (P01, Hospital, Physician, Germany)*

*‘We have a great many training courses that we can offer to family caregivers. Yes, of course, this allows for longer care at home in a domestic setting.’ (P06, Long-term care, Nurse, Germany)*

Additionally, they declared that patients and their caregivers have access not only to education and information regarding medical aspects but also administrative and organizational. Information and courses are provided in the care advice centers that are widely available. Nonetheless, information and education are also provided by the providers, for instance, in the form of regular meetings with the patients and their families as one participant suggested. On the other hand, according to informants in Germany, one of the major barriers that could affect care transitions is the fact that patients’ and caregivers’ needs, and preferences are not considered.

*‘…because the patient’s needs are not considered. So there are other mechanisms behind the decisions, whether a patient is transitioned or not.’ (P03, Primary care, Nurse, Germany)*

Moreover, participants argued that patients and their caregivers are often not involved in the decision-making process. One informant added to that by arguing that caregivers’ in Germany tend to be poorly involved and informed and therefore, might have limited knowledge on how to care for or assist older adults. For this reason, some participants stated that it is crucial to involve patients and caregivers in the care process.

*‘…every person is self-determined, i.e. he has a fundamental right to be involved in everything that concerns him and to be able to make decisions about it.’ (P08, Payer/Insurer, Management, Germany)*

**Sub-theme 6: Telemedicine and e-Health**

Nearly all informants agreed that the use of telemedicine and e-Health is very limited in Germany.

*‘We are still in the real beginning in Germany with the implementation of e-Health’ (P03, Primary care, Nurse, Germany)*

One participant suggested that limited use of telemedicine might negatively affect care transitions by, for example, delaying patients’ discharge to home. In their view, telemedicine and e-Health should be utilized more as it has the potential to optimize care transitions by, for instance, improving the transfer of information. Specifically, half of the participants expressed the need for electronic patient records for information access and transfer.

*‘I have to say, if we had a digital transfer of patient files. That would make it a lot easier.’ (P06, Long-term care, Nurse, Germany)*

Moreover, some informants also acknowledged the important role of video consultations and tele-nursing and health monitoring devices.

*‘If you talk about video consultations […] this is a very good option to keep them at home, to keep them away from the risks that they have in hospital.’ (P01, Hospital, Physician, Germany)*

One participant declared that video consultations are slowly being introduced in Germany. Nevertheless, two informants argued that some older adults, especially those cognitively impaired, might lack knowledge on how to use telemedicine and/or e-Health. Additionally, they suggested that some health professionals might be reluctant to use telemedicine due to the heavy workload in their clinics.

**Sub-theme 7: Social care**

According to the informants, social care institutions play an important role in optimizing care transitions in Germany by helping the patient and/or caregiver to cover the LTC costs if they are incapable of paying.

*‘The social welfare office always kicks in when the patient can no longer afford to pay (for LTC).’ (P07, Payer/Insurer, Management, Germany)*

They suggested that social care institutions help to cover the costs of LTC without any issues. On the contrary, one participant declared that social care institutions are sometimes unwilling to cover the LTC costs, especially if they rise. Moreover, informants had unambiguous opinions regarding the role of social care workers in inpatient settings. Some of them argued that social care workers play an important role in discharge management, for example, by preparing the receiving setting. At the same time, others had mixed feelings regarding their involvement in discharge planning.

*‘I am not aware of convincing literature that certain structures or that we need social care workers for...discharge planning.’ (P03, Primary care, Nurse, Germany)*

One participant added to that by arguing that having the separate role of social care worker might lead to diffusion of responsibilities.

*‘…and what I sometimes find, especially in hospitals, is that there is a diffusion of responsibility.’ (P02, Hospital, Nurse, Germany)*

Moreover, the role of social care workers in outpatient settings is very limited, as suggested by one informant.

*‘So in the in the in the outpatient setting, they have no impact I would say in Germany.’ (P03, Primary care, Nurse, Germany)*

Furthermore, participants argued that they are difficulties in communicating with social care institutions experienced not only by the providers but also by the patients and their caregivers. Nonetheless, according to informants, it is very important to involve social care institutions/workers in interprofessional and intersectoral meetings.

*‘…and if we have successful case conferences, then of course, it's very important to have social workers there.’ (P02, Hospital, Nurse, Germany)*

One participant stated that social care workers are already involved in regular meetings with providers.

**Sub-theme 8: Supporting informal caregivers**

Nearly all informants in Germany agreed that there is vast availability of training courses for informal caregivers.

*‘We have great many training courses that we can offer to family caregivers.’ (P06, Long-term care, Nurse, Germany)*

Moreover, they reported also that informal caregivers have access to different information centers providing advice. Nonetheless, in their opinion, sometimes these courses and information centers are not attended by informal caregivers. Furthermore, informants argued that informal caregivers are neither sufficiently involved and informed, nor receive sufficient support during the care transition.

*‘So the involvement of informal carers or relatives, in general, is one of the most negative things I experience here […] but we tend rather have them (informal caregivers) not informed […] and the avoidance of unnecessary or maybe too late transitions also depends on what they know.’ (P01, Hospital, Physician, Germany)*

One participant argued that family’s determination might also limit the amount of support and information they receive. On the other hand, informants suggested that informal caregivers can access available respite care services, receive financial compensation for providing care and get a pension covered by the insurer. Nonetheless, it is essential to provide information regarding institutions and professionals that offer such help and respite care, as one participant suggested. Additionally, some informants stated that there is a need to increase the budget for respite care so that caregivers might take more days off to rest and, thus, continue providing care for longer.

*‘I think this should be expanded in order to offer more relief to the caring relatives.’ (P06, Long-term care, Nurse, Germany)*

At last, participants argued that there is a need for assessment of informal caregivers’ needs and for provision of training to caregivers teaching them how to take care of themselves since they currently receive limited psychological support.

**Financial challenges**

The knowledge of some participants regarding financial aspects was limited and for this reason, some informants were unfamiliar with the impact of reimbursement, rewards and penalties on care transition. Nevertheless, informants in Germany agreed that particularly reimbursement-related factors might have an immense impact on the care transition of older adults.

**Sub-theme 9: Reimbursement**

The role of reimbursement and its impact on care transition was one of the most discussed subjects among German informants. Nearly all participants agreed that out-of-pocket payments are one of the most important factors affecting the care transitions of older adults in Germany.

*‘The personal contribution that the person in need of care has to make is often decisive here.’ (P08, Payer/Insurer, Management, Germany)*

They argued that patients’ and caregivers’ possibility to access LTC might be restricted due to their inability or unwillingness to cover high out-of-pocket costs.

*‘And it's an argument that you can't go into a nursing home because the relatives have to pay for it themselves in the end through social insurance if your own assets are not sufficient.’ (P04, Long-term care, Nurse, Germany)*

Fortunately, in such situations when patients and their caregivers are unable to pay, social care institutions step in to help to cover the costs of LTC placement, as stated by the informants.

*‘And when the funds have been used up […] we have to contact the social welfare offices […] luckily, they will step in and assume the costs of inpatient care.’ (P06, Long-term care, Nurse, Germany)*

On the other hand, one participant suggested that social care institutions are sometimes unwilling or hesitant to pay to support LTC placements. For this reason, increasing public funding for LTC could help to reduce out-of-pocket payments, and as a result, lead to the optimization of care transitions, as stated by one informant. Furthermore, informants argued also that lack of reimbursement for interprofessional collaboration/intersectoral care/transitional care might negatively affect care transitions. Besides that, informants discussed different payment mechanisms and their impact on care transition. In their opinion, payments per-diem in Germany might lead to suboptimal care transitions by inclining providers to reduce staff and admit healthier patients (cream-skimming).

*‘Flat-rate systems lead to a certain distortion here.’ (P08, Payer/Insurer, Management, Germany)*

Moreover, one participant blamed current DRG reimbursements in hospitals for shortening the length of stay without justified cause and thus, leading to suboptimal care transitions.

*‘…we often see patients being discharged from the hospital too quickly with the message that the patient is cured or that we can no longer expect any improvement but, in our opinion, a hospital stay for 2-4 days would have done the patient good.’ (P06, Long-term care, Nurse, Germany)*

Besides that, informants argued that activity-based payments might result, for instance, in supplier-induced demand and ultimately negatively affect care transitions. Value-based payment methods were also questioned by the participants, and the difficulties in measuring the quality of care were acknowledged. One of the informants was in favor of evidence-based reimbursements and simply - reimbursing what has proven to be effective.

*‘…one of my wishes is that reimbursement mechanisms better follow...the evidence about what works for patients.’ (P03, Primary care, Nurse, Germany)*

Another mentioned that to optimize care transitions and to reduce barriers between the settings - different settings should be financed by single-payer.

*‘If the financing is one hand, then you have fewer barriers between the settings. For example, you have less interest, financial interests and…and probably have more…more time to plan transitions.’ (P02, Hospital, Nurse, Germany)*

At last, some informants argued that current reimbursement rates for LTC facilities and salaries for staff are sufficient and satisfactory, while others questioned the insufficient reimbursement levels for the care provided. According to their opinion, additional mechanisms, such as lump sums, should be introduced to compensate for variability in incurred costs. Lump sum payments should also be introduced for the transition period before patients’ disability score is estimated since, according to informants, delays in providing disability scores influence providers’ reimbursement and thus might lead to suboptimal care transitions.

*‘For example, if we are offered a patient by the hospital who has not yet been assigned a degree of care […] then we often refrain from admitting this patient’ (P06, Long-term care, Nurse, Germany)*

At last, participants suggested that availability of LTC insurance, reimbursement of video consultations and training for informal caregivers could result in the optimization of care transitions.

**Sub-theme 10: Rewards**

Some of the answers provided by the informants on this theme were vague or incomplete, as some of participants had limited knowledge regarding rewards.

‘*I don't know, actually so […] I don't think I can make a good statement on this. Sorry.’ (P01, Hospital, Physician, Germany)*

Nonetheless, some informants argued that there are no rewards for providers in the German health and social system. Moreover, they rather had mixed feelings or even felt hesitant about the use of rewards.

*‘I think I'm not convinced that these (rewards) are the driving factors.’ (P03, Primary care, Nurse, Germany)*

On the other hand, some participants argued that rewards might potentially improve care transitions or stimulate practices. However, they were unable to explain how it could be achieved. One informant stated that increasing salaries for the staff would be better stimulus than rewards. Furthermore, while discussing the role of rewards and their impact on care transitions, some participants raised an issue of measuring the quality of care and thus, appointing those eligible for the reward. Besides that, informants also questioned whether rewards are effective in the long term and pinpointed the problem of fraud where providers try to ‘cheat’ the system by ‘pretending’ that criteria are met.

*‘What I frequently see in the system is that… the system is usually smarter than the criteria so then even the ones who don’t get it right now the criteria, and they at least pretend to get it right.’ (P02, Hospital, Nurse, Germany)*

**Sub-theme 11: Penalties**

The issues raised within the theme penalties were to some extend similar to those of rewards. According to German informants, penalties are not available in the German health and social system. Their opinions regarding the use of penalties are rather diverse. Some participants expressed their mixed feelings or even hesitancy about the use of penalties, while others consider penalties as an opportunity to raise awareness about the problem or punish providers for different kinds of abuse, misuse, and abnormalities.

*‘I would be happy if you could get the money back for the mistake and a penalty on top of that.’ (P07, Payer/Insurer, Management, Germany)*

Nonetheless, one informant argued that penalties on their own are insufficient measures. They should be constructive and offer solutions. Besides that, one participant argued that too minor penalties might not have the desired effect.

*‘I think it's a matter of the opportunity cost […] because one question is how much are those affected by the penalties that will be enacted?.’ (P03, Primary care, Nurse, Germany)*

At last, informants discussed issues related to measuring quality of care and appointing a responsible party if something goes wrong.

*‘How can you really prove that a hospital released the patient too early? I imagine that's very difficult’ (P06, Long-term care, Nurse, Germany)*

**Appendix 4. Key findings – The Netherlands**

Semi-structured interviews with 8 key country informants enabled us to identify important sub-themes for organizational and financial themes affecting care transitions of older adults in the Netherlands. We interviewed following participants: providers - 1 from primary care, 2 from hospital, 4 from long-term care, and 1 insurer/payer.

Similarly, to Poland and Germany, majority of the experts agreed that currently, care transitions in the Dutch long-term care system are not optimal and there is a room for improvement. Participants argued that broader organizational and financial aspects might affect not only the quality of care transition but also the direction. Below we present different organizational and financial aspects that affect care transition in the Dutch long-term care system.

**Organizational challenges**

Country informants in the Netherlands agreed that organizational aspects, particularly, communication and transfer of information have an immense impact on care transition of older adults. In their view, current communication and transfer of information is not optimal and there is still a lot to be improved if the Netherlands is to deliver safe and seamless care transitions for older adults.

**Sub-theme 1: Communication**

According to majority of experts, good interprofessional/intersectoral communication among all providers/institutions involved in the care process is essential for optimal care transitions. One participant argued that such communication should include discussion about the patients’ medical, psychological, social and caring needs. Others suggested that current interprofessional/intersectoral communication, particularly between the hospital and home care, is not always optimal and that there is a need for improvement.

*‘But you have some communication issues between the care providers from the hospital to home.’ (P10, Hospital, Nurse, The Netherlands)*

Participants suggested that especially the availability of multidisciplinary team meetings improve communication as it enables the professionals, organizations, and institutions to get to know each other, their roles and expertise. They argued that knowing professionals from other organizations/institutions personally might ease communication. One expert also suggested that organizations/institutions affiliated with hospitals communicate easier and better.

*‘We know that there are institutions that are part of the hospital and of course then the the, it's quite easy for...to transfer between the institutions and the communication is a lot better and it's easier.’ (P16, Payer/Insurer, Management, The Netherlands)*

Besides that, participants recognize the importance of good communication with the patient and informal caregiver.

*‘We take the time to have a conversation with them (the patients and informal caregivers) that they feel heard.’ (P13,14, Long-term care, Nurse, The Netherlands)*

One expert stated that professionals from long-term care facilities contact the person at home to get acquainted with the patient and their informal caregiver.

*‘The location (long-term care facility) will come to the person in the home situation so they can get acquainted.’ (P11, Primary care, Management, The Netherlands)*

Furthermore, district nurses might also visit and communicate in person with the staff at the LTC institutions as suggested by an expert. Moreover, professionals such as community nurse, transfer nurse and social care workers are responsible for communication with the LTC institutions or community care and arranging the place for the patient. One participant stated that current communication between home care and LTC care institutions is well organized.

*‘But I think the professionals communicate very well... So, for example, we have a patient in the home care and he needs to be admitted at one of the long term facilities, the nurses that are working in home care they contact the location.’ (P11, Primary care, Management, The Netherlands)*

Besides that, participants declared that the use of video calls, telephone calls and in general e-Health solutions might improve the communication not only between the providers but also with the informal caregivers.

*‘Being able to communicate with nurse via the app is quite helpful.’ (P16, Payer/Insurer, Management, The Netherlands)*

Sub-theme 2: Transfer of information

Transfer of information is another important organizational aspect that might affect care transition in long-term care systems. Dutch experts seem to agree that good transfer of information between the providers/institutions, the patient and informal caregivers is essential for optimized care transition.

*‘I think it's important...Yeah. You need to have the right information.’ (P11, Primary care, Management, The Netherlands)*

According to their opinion, current transfer of information is not always optimal. One participant argued even that transfer of information is one of the biggest flaws in the Dutch system.

*‘That's (transfer of information) one of the big flaws, I think…’ (P09, Hospital, Nurse, The Netherlands)*

Participants argued that in some cases transferred information is lacking details, is incomplete and/or delayed.

*‘Information transfers are often lacking or too late or incomplete.’ (P09, Hospital, Nurse, The Netherlands)*

They suggested that transferred information sometimes includes only one providers’ perspective and does not contain psychological aspects. On the other hand, including not only medical but also psychological and social aspects was considered by Dutch participants as important factor in optimizing information exchange. According to experts not only quality of transferred information but also its timeliness are important factors for smooth care transitions. Besides that, participants declared that factors such as the availability of agreements between the providers/institutions and, in general, interprofessional collaboration could improve transfer of information.

*‘We know that there are institutions that are part of the hospital and of course then, it's quite easy for...to transfer between the institutions and the communication is a lot better and it's easier to give the information.’ (P16, Payer/Insurer, Management, The Netherlands)*

Experts suggested that it is also very important to have an integrated platform, for instance in form of electronic patient record, where information could be exchanged between the providers/institutions.

*‘If we can have electronic files about the patient, it does help because then it's easier for different caregivers to to find out about the the elderly and what its ailments are.’ (P16, Payer/Insurer, Management, The Netherlands)*

Some participants complained that currently there is lack of integrated system for information exchange and every provider has their own system. Moreover, in their view, standardized protocol for information exchange might improve transfer of information, and ultimately care transitions. Other factors that could have an impact on suboptimal transfer of information refer to the privacy laws that might restrict transfer of information between the institutions and lack of participation of the provider in digital solutions to transfer information.

*‘Sometimes it's just the the laws. So the the documents that we have, we're not allowed to transform from one institution to another.’ (P16, Payer/Insurer, Management, The Netherlands)*

**Sub-theme 3: availability & coordination of resources**

Majority of experts in the Netherlands seem to agree that there is a need for good interprofessional and intersectoral collaboration among all involved in the care process.

*‘I think it's important that all care providers are involved when talking about care transition.’ (P10, Hospital, Nurse, The Netherlands)*

According to participants, providers/institutions tend to work in silos and there is lack of collaboration between them.

*‘At the moment in the Netherlands disciplines in community care are working pretty much solistic, so nurses, community nurses work for themselves, community physical therapists work for themselves and so on.’ (P09, Hospital, Nurse, The Netherlands)*

Experts argued that support from the management is essential to improve interprofessional/intersectoral collaboration. Moreover, they suggested that the availability of agreements between the providers and institutions might also have a positive impact on interprofessional/intersectoral collaboration, and ultimately care transitions.

*‘Well, I think that for certain regions, where there are agreements between organizations, that definitely makes it easier to transfer a patient.’ (P15, Long-term care, Physcian, The Netherlands)*

Additionally, experts argued that there is a need for clear definition of responsibilities of professionals and organizations.

*‘If we don't say that's part of your job and you need to do that, it's yeah. I think it's not going to, going to work.’ (P11, Primary care, Management, The Netherlands)*

One participant stated that especially in large organizations responsibilities are sometimes not clear and thus, fragmentation of care might occur. Nevertheless, according to some experts, having multidisciplinary team meetings not only in the care setting but also in the community might reduce care fragmentation. One participant added to that by suggesting the importance of embedding multidisciplinary team meetings in routine care.

*‘So if it's routine care, having this interdisciplinary multidisciplinary collaboration […] and so the routine of doing so is very important.’ (P09, Hospital, Nurse, The Netherlands)*

Currently, such interdisciplinary team meetings are taking place in their organization as stated by an expert.

*‘At the neurosurgery department, we have very strict organized multidisciplinary team meetings.’ (P09, Hospital, Nurse, The Netherlands)*

Furthermore, according to one participant, transitional care interventions with the use of already existing care networks should be also embedded in routine care. Additionally, some of the transitional care interventions should consider home as a starting point so that care transitions are prevented from the first place. Besides that, experts pointed to the important role of different professionals in care transition, specifically, involvement of physiotherapists, transfer nurses, community nurses and care transition managers in the LTC settings. For instance, transfer nurse in the hospitals play an important role in care transition by preparing and communicating with the family and receiving setting.

*‘The transfer nurse is the one that talks to the family, to the relatives that investigates which...institution the elderly can go to, and also is the one that informs the institution.’ (P16, Payer/Insurer, Management, The Netherlands)*

On the other hand, participants discussed an important role of the LTC infrastructure and its impact on care transition. They argued that aspects such as the availability of staff, number and location of LTC institutions, and the availability of crisis beds in the nursing homes might impact care transitions. According to the participants, there is an increasing problem with the availability of staff and, in some cases, waiting time to access the next setting, particularly LTC institutions.

*‘It's difficult to have enough staff within the community care services at the moment.’ (P09, Hospital, Nurse, The Netherlands)*

Another problem is obtaining Wlz indication that enables the patient to receive LTC either in either LTC institutions or care at home provided by the professionals. Experts suggested that criteria for obtaining Wlz indication is strict and not rational and that the waiting time for obtaining the indication might lead to suboptimal care transitions. As a result, participants argued about the importance of patients’ assessment and the timely and right indication.

At last, experts also argued that performing advanced care planning, transition planning and making it accessible to all providers is crucial to ensure smooth care transition process.

*‘[..] and with a good plan, not for only this transition, but further on to work […] but there needs to be a plan for the whole trajectory of the patient.’ (P09, Hospital, Nurse, The Netherlands)*

**Sub-theme 4: training and education of staff**

One expert believed that the staff in the Netherlands is well trained and educated and that there is no need for extra education or training.

*‘I think they know what is important. They don't need extra education to...to service a good transition. No, I don't think they need that.’ (P11, Primary care, Management, The Netherlands)*

On the other hand, some participants suggested that there is still a room for improvement.

They argued that some professionals lack basic knowledge about how the care is organized among other professions, and/or in other settings, and thus don’t know about possible help or advice they could receive from each other.

*‘And my experience is that many caregivers, professionals don't even know what the other profession does or what's available.’ (P09, Hospital, Nurse, The Netherlands)*

According to experts, it is important that professionals understand the work of other providers/institutions and look at care from different perspectives.

*‘But it is important to be able to look over your own profession and to know what the other does, and also to to know how important that can be for your patient.’ (P09, Hospital, Nurse, The Netherlands)*

To address this gap, experts suggested that staff should be made aware or even trained about work of different professionals, and other settings. Some participants argued that this could be achieved with the use of multidisciplinary team meetings.

*‘That could be so interactive like in, in an organized setting from an multidisciplinary team meeting that, that's, that's I think an important basis where also students and new colleagues enter and they get to know each other as well.’ (P09, Hospital, Nurse, The Netherlands)*

Moreover, one expert suggested that some staff in the community settings have rather generic education and might have difficulty in dealing with complex patients, for instance, with specific diseases. Therefore, this participant suggested that educating the staff to recognize some disease specific vital signs is important for optimized care transitions. Besides that, some experts argued that education regarding transitional care should be an essential part of each education program.

*‘They need to know what is important to do when there is a transition. So I think that's an essential part of each educational program.’ (P15, Long-term care, Physician, The Netherlands)*

Furthermore, one expert argued that it is important to provide staff with additional trainings, for instance, on “soft landing” or regarding information exchange to improve quality of care transitions. At last, one expert argued that is it important to change the mindset of professionals from “taking over” care from the patient and informal caregiver to more interaction and support-based model where staff is educated and aware about the important role of providing support for self-management.

*‘We have to, we have to educate people at that because we want them to act different, because we want them to support and to to... how you say it...supporting...and to stimulate the people who are in demand of care to do things primarily themselves.’ (P12, Long-term care, Management, The Netherlands)*

**Sub-theme 5: education and involvement of the patient and/or caregiver**

Nearly all experts agreed that well-educated and informed patient and informal caregiver are one of the key components for optimized care transitions.

*‘[..] because if the client or the family are rightly informed, they know what is gonna happen and they know what they need to do.’ (P13,14, Long-term care, Nurse, The Netherlands )*

According to participants, to achieve it, it is necessary to provide multidimensional information/education to the patient and caregiver at an early stage. Currently, such information/education is already provided to the patient and their informal caregiver as indicated by some experts.

*‘We provide them (the patient and the informal caregiver) information in in in the early stage.’ (P13,14, Long-term care, Nurse, The Netherlands)*

Nevertheless, one expert argued that provided information/education might vary among providers/institutions.

*‘It really depends on the hospital, or the nursing home or the doctor, whether he or she is willing to inform.’ (P16, Payer/Insurer, Management, The Netherlands)*

Besides that, in their view, to optimize care transitions, it is important to consider patients’ and caregivers’ needs and preferences and to involve patient and caregiver in decision-making process.

*‘Optimal care transition is a care transition that's according to the needs and preferences of the patient.’ (P15, Long-term care, Physcian, The Netherlands)*

One expert claimed that current care is not always patient-centered.

*‘I think we're we're getting better at it, but we're not putting the client, as we call it, the patient, the elderly as focus point.’ (P16, Payer/Insurer, Management, The Netherlands)*

In addition, experts discussed an important role of informal caregivers and their involvement in the care process. According to their opinion, informal caregivers should be actively involved as they have the closest contact with the patient. Nonetheless, some participants argued that in some cases, informal caregivers are either not involved or insufficiently involved in the care process.

*‘But in general, as to my perspective…there's no structural way families are involved in most settings. And if they are not there at the moment, the physicians won't talk to them.’ (P09, Hospital, Nurse, The Netherlands)*

This is particularly a problem when the patient moves to long-term care facility as suggested by one expert. Besides that, participants suggested that it is important not only to assess informal caregivers’ ability to provide care, but also to address psychological needs of the patient and informal caregiver.

**Sub-theme 6: telemedicine and e-Health**

According to Dutch experts, e-Health and telemedicine plays an important role in optimizing care transitions of older adults.

*‘I think e-Health is very important.’ (P12, Long-term care, Management, The Netherlands)*

Nevertheless, one participant expressed their mixed feeling regarding the use of telemedicine and its effectiveness. One the other hand, participants argued about the important role of electronic devices to monitor patients at home and the use of telemedicine in self-management. According to them, telemedicine might help the patient to be more independent and improve communication, particularly with informal caregivers.

*‘So, for example, if they have the medication in the system, the nurses don't have to come every day to give them the medicine. They can do it themselves as long as they get a little alarm.’ (P11, Primary care, Management, The Netherlands)*

As an example, one expert discussed the use of medication dispenser by the patient at home, but there are also other examples of the use of telemedicine in the Dutch long-term care system. However, one participant argued that the use of telemedicine stops once the patient moves to the long-term care facility. Besides that, experts argued about the important role and the need for electronic patient record that would be accessible to all providers.

*‘If we can have electronic files about the patient, it does help because then it's easier for different caregivers to to find out about the elderly and what its ailments are.’ (P16, Payer/Insurer, Management, The Netherlands)*

Nonetheless, to make the use of telemedicine and e-Health successful, participants suggested that it is important to provide resources in terms of devices etc. to the patient and the staff as they might not be able to afford it. Moreover, they argued that there is a need for personalizing telemedicine and e-Health solutions to better address the patient’s needs. At last, experts argued that factors such as privacy issues, complexity of needs of older adult patients and lack of integration of the providers in digital solutions might further limit the use of telemedicine and e-Health.

*‘Many of our frail elderly have multimorbidity and not only physical problems, but also functional limitations, psychological, social aspects. And I think that will be difficult to cover all of that with tele-health.’ (P15, Long-term care, Physician, The Netherlands)*

**Sub-theme 7: social care**

According to few experts, social care workers play important role in care transitions.

*‘Well, in our organization, they (social care workers) are essential. They have a major role.’ (P15, Long-term care, Physician, The Netherlands)*

Currently, social care workers are often engaged in preparing the transitions from the hospitals and home care to nursing home. Participants declared that their involvement in the hospitals and home care is important as social care workers are well informed about different organizations providing care. Moreover, experts suggested that social care workers have more time to look at aspects beyond medical care and thus, know the patients’ needs and preferences, their environment and therefore, can assess the patients’ situation holistically.

*‘[…] social workers to have more time for the psychological guidance and…are also able, I think, to have more time to go into it, to ask for in-depth information.’ (P09, Hospital, Nurse, The Netherlands)*

*‘I think social workers have more view on what's really needed for the whole situation.’ (P09, Hospital, Nurse, The Netherlands)*

Besides that, participants argued that social care workers play important role in providing support and assistance to the patient and informal caregiver and could potentially focus on patients activization through social engagement.

*‘So...social workers can be there very important because they can go to people or they can say, okay, I'm coming to you and we’re going for a walk, or we're going to a society for a cup of coffee […] to take people out and to give them attention. And I think that that is also part of the solution […] to be less dependent and be more actively.’ (P12, Long-term care, Management, The Netherlands)*

Nonetheless, social care workers are not always involved in care transitions, as stated by one participant. Moreover, one expert claimed that social components are not addressed enough.

*‘So we do that, I think, but not enough.’ (P11, Primary care, Management, The Netherlands)*

Besides that, some participants questioned the role of social care workers and its’ impact on care transition. According to them, the role of social care worker could be performed by other professionals, for instance, nurse. One expert argued that a district nurse knows the patient better than social care worker.

**Sub-theme 8: supporting informal caregivers**

There is no consensus among Dutch experts whether current support provided to informal caregivers is sufficient. On one hand, some participants argued that provided support is sufficient as professionals provide informal caregivers not only with information, guidance, and support but also, they bring them in contact with the right professionals. In addition, there are organizations that provide support to informal caregivers as stated by one expert.

*‘Yeah, we have to organize that (support for informal caregivers). I have just said, we are working closely with an organization for informal caregivers. So that means that we give instruction, we guide, and we support them.’ (P12, Long-term care, Management, The Netherlands)*

On the other hand, some participants suggested that informal caregivers do not receive enough support during care transition, and that the support provided varies among organizations/institutions.

*‘I don't think so. I don't think, well, maybe depends on the organization you're talking about.’ (P09, Hospital, Nurse, The Netherlands)*

Moreover, one participant stated that there is lack of structural involvement of informal caregivers in most settings. Besides that, according to experts, it is essential to assess caregivers’ needs and to provide psychological and social support they might need.

*‘[…] but also because of the risk of caregiver burden. I think it's also important that the community nurse, for example […] offer some psychological or social support.’ (P10, Hospital, Nurse, The Netherlands)*

**Financial challenges**

The knowledge of some participants regarding financial aspects was limited and for this reason some experts were unfamiliar with either impact of reimbursement, rewards and penalties on care transition. Nevertheless, experts in the Netherlands seem to agree that particularly reimbursement related factors seem to have an immense impact on care transition of older adults.

**Sub-theme 9: reimbursement**

Some participants had limited knowledge regarding the impact of reimbursement on care transitions. Nevertheless, according to experts in the Netherlands, out-of-pocket payments for LTC might have an impact on care transitions of older adults. As a result, some patients might be reluctant, for instance, to move to institutions as it is more expensive for the patient than staying at home.

*‘Well, I think there might be situations where the amount that patients have to pay...for care can be a problem.’ (P15, Long-term care, Physician, The Netherlands)*

Moreover, some individuals might be hesitant in obtaining Wlz indication due to associated costs.

*‘Some clients don’t think it's it's needed that they have an indication because they need to pay their own insurance about that indication. And that can be a big amount of money.’ (P12, Long-term care, Management, The Netherlands)*

Besides that, participants argued about the importance of sufficient reimbursement level of providers/institutions and the importance of satisfactory salaries for staff. In addition, some experts suggested that current reimbursement of the providers is not optimal and might result in financial loss for the organizations. This is particularly connected with the fixed reimbursement per patient according to indication, irrespective of variability in care needs. As a result, the organization receives the budget, and the nurses receive salary independent of volume of care provided. Low salaries for LTC staff, particularly community nurses, and low financial resources for LTC are another issue that were discussed by the experts that might indirectly affect care transitions.

‘*Well they, their (community nurses) wages are quite low, and so it's not really attractive to work in…’ (P09, Hospital, Nurse, The Netherlands)*

Participants claimed that low financial resources for LTC have an implication on the availability of beds in LTC and staffing levels, and thus there is a need for increasing financing for LTC to enable smooth transition between the settings. Specifically, the reform in 2015 and cutting the budget for long-term care had an impact on number of LTC settings as stated by one participant. Besides that, experts spoke in favor of introducing reimbursement for interprofessional/intersectoral/transitional care collaboration. Currently, no such reimbursement exist. Participants argued that this is specifically the problem when providers from other settings (e.g. long-term care facility, home care) want to visit the patient in another setting (e.g. hospital)

*‘For example, we have a case managers dementie (dutch, dementia) […] They help people with dementia and when somebody that has dementia is located to a hospital or to a long-term facility, they wish to visit the patient in the other setting. But if they do that […] we cannot get a finance for that.’ (P11, Primary care, Management, The Netherlands)*

Experts spoke also about the role of activity- and value-based payments. Some participants had mixed feelings regarding the activity-based payments and value-based payments. According to their opinion, activity-based payments could have negative impact on care transition by, for instance, leading to overproduction. On the other hand, some experts suggested that value-based payment methods might have potential to improve quality of care while at the same time acknowledging the difficulties in measuring quality of care. According to one participant, extra quality reimbursement as a part of standard reimbursement has been introduced some time ago, however, the effects are still unclear. At last, experts argued about the importance of including community nurses and physiotherapist in basic insurance package. Currently, physiotherapy is not included in the basic insurance and that should change if the aim is to optimize care transitions as stated by one participant.

*‘And from the physical therapist what I told you this is not in the regular insurance. So patients have to pay more to have physical therapists in their insurance.’ (P10, Hospital, Nurse, The Netherlands)*

**Sub-theme 10: rewards**

Dutch experts had mainly mixed or even negative feelings towards the use of financial rewards. According to their opinion, internal motivations of staff to provide good quality care is more important than financial rewards.

*‘I think that the motivation for transitional care could be internal because health care providers would like to provide good care to patients.’ (P10, Hospital, Nurse, The Netherlands)*

On the other hand, some participants argued that financial rewards at the organizational level could potentially improve quality of care transitions, for instance, by encouraging collaboration between professionals/providers/sectors. Moreover, one expert suggested that financial rewards could be potentially reinvested by the organization to further improve quality of care by investing for example in education of the staff. As a result, one participant claimed that rewarding organizations once a year based on their performance could be introduced as it would be additional stimulus for the organizations/institutions to improve collaboration.

*‘I think that would work if…for instance, on a yearly basis one one evaluates how care transfers were performed […] and if we are able to reduce that waiting time and have smooth transfers and you get rewarded, yeah, that works of course, because then there's an impulse to even more talk with the rehabilitation centers on how to improve the transfers.’ (P09, Hospital, Nurse, The Netherlands)*

Besides that, one participant argued that system of rewards was implemented in the Netherlands, however, in their view, financial rewards are short term stimulation and once the reward stops, the efforts to improve quality of care also stop.

*‘We did work with a reward system sometimes, but then when you stop the reward, it's...the extra stops as well. So it's, it's a short term gain.’ (P16, Payer/Insurer, Management, The Netherlands)*

In addition, expert claimed that having rewards for long-term is not possible as someone would need to pay for it. On the other hand, participant suggested that providing financial means to the providers so that they can improve competencies of the staff is better solution since the effect is maintained.

**Sub-theme 11: penalties**

Experts mainly had mixed feelings or even negative feelings regarding the use of penalties and its impact on care transitions of older adults. According to some participants, there are already penalties for the providers in the Dutch system, however, not for community nurses. One expert mentioned the availability of benchmarking and receiving less or more money based on providers’ performance.

*‘I already think there are penalties because you have like a benchmark […] so we have to get some kind of a score to get good finance. So there already is a negative or a positive influence on the financial statement.’ (P11, Primary care, Management, The Netherlands)*

Another participant argued that penalties could be issued for inappropriate care, referral, bad communication, transfer of information or delayed care. On the other hand, some experts argued that the penalties do not work or that their effect is short-lived.

*‘It does help, but it only helps for a very short period of time and an incentive is just a year or two years, whatever, and then you get used to it and it doesn't work anymore.’ (P16, Payer/Insurer, Management, The Netherlands)*

Besides that, one participant suggested that penalties could be even harmful and negatively affect quality of care by further restraining the budgets for LTC providers.

*‘We, in the long-term care, the government says that we should punish...institutions that have poor quality of care. I'm dead against it, because if you take away the money, the quality will not improve. It will only get worse.’ (P16, Payer/Insurer, Management, The Netherlands)*

One expert mentioned also that the availability of penalties could have an impact on wider policy context while specifically referring to admission policy and admitting healthier patients as an example. Furthermore, participants discussed problems with measuring the quality of care, appointing responsible party if something goes wrong and providers that try to “cheat” the system just to avoid the penalty. At last, some participant argued that internal motivations of staff to provide good care is more important than financial penalties.

**Appendix 5. Key findings – Poland**

Semi-structured interviews with 7 key country informants enabled us to identify important sub-themes for organizational and financial themes affecting care transitions of older adults in Poland. We interviewed following participants: providers - 2 from primary care, 2 from hospital, 2 from long-term care, and 1 insurer/payer.

Most of the informants agreed that care transitions in the Polish long-term care system are not optimal and still, a lot has to be done if Poland is to deliver safe and seamless care transitions for older adults. Participants argued that broader organizational and financial aspects may affect not only the quality of care transition but also the direction. Below we present different organizational and financial aspects that affect care transition in the Polish long-term care system.

**Organizational challenges**

Country informants in Poland suggested that organizational aspects, particularly, communication, transfer of information and coordination of resources play an important role in care transitions of older adults. In their view, addressing all these aspects is necessary in order to optimize care transitions in the Polish long-term care system.

**Sub-theme 1: Communication**

All participants agreed that the communication between providers is very limited or even non-existent and that there is a need for better communication, particularly timely communication between sending-receiving settings. Moreover, participants suggested that providers do not use methods of direct communication and rarely communicate with each other in order to organize the care for the patient.

*‘There should be communication with the entity (organization) […] and the patient should immediately go to such a place, and this is not the case’ (P22, Long-term care, Management, Poland)*

*‘There is no single form of handing over a patient […] if patients call me earlier, they have everything taken care of when the patient comes home, and if they do not have such information, then he lies on a couch for several days and already has pressure ulcers’ (P19, Primary care, Nurse, Poland)*

According to participants, social care workers at the hospital are among the few that communicate with other providers (particularly long-term care facilities), and therefore, their role is considered important among informants. Some respondents suggested that telephone calls shall be introduced to communicate with patients and/or caregivers and to monitor patients’ health status.

*‘This system of communication with people, with seniors, well, the simplest is the telephone, right? Because as I said one during the conversation, you can tell if the patient is not confused or has memory problems […] or taken pills, well a simple set of questions’ (P23, Payer/Insurer, Management, Poland)*

Moreover, participants highlighted that communication of the providers with the patient and/or family is also very limited and needs to be improved as it leads to suboptimal care transitions.

*‘The ambulance arrived, discharged from the hospital and they came with the patient to the door […] the son knew that his mother was in the hospital, they did not let him know that his mother would be leaving […] and what did the ambulance have to do is to drive the woman back to the hospital, because they wouldn't leave her in the middle of the road’ (P19, Primary care, Nurse, Poland)*

**Sub-theme 2: Transfer of information**

Transfer of information is another important organizational aspect that may affect care transition in long-term care systems. Some participants declared that the transfer of information between providers is very limited, unstructured, and often incomplete, particularly between hospital and primary care. According to some, this could be the result of a lack of legal regulation regarding the transfer of information. Moreover, participants argued that the use of the available online platform to transfer information is very limited and rather outdated methods of transferring information, such as, on paper, are used. Additionally, participants expressed their concern regarding the responsibility of the patient to carry the information and deliver it to the following setting.

*‘He (patient/caregiver) gets an information card in the corridor and information that tomorrow at 3 pm we will discharge the patient and that's it.’ (P21, Long-term care, Management, Poland)*

Since the patient carries the information, providers lack direct contact with each other.

According to country informants, the problem with the transfer of information also takes place within the settings. Participants suggest that there is a need to improve information exchange between different providers so that the receiving setting can get prepared to address patients’ needs.

*‘….whether the patient is tube fed, has a catheter or no catheter. Such simple things, although it is true, would make things much easier, I think, for both parties. I also think that the transfer of information is very important, and yet it escapes somewhere and is practically not in our care, in our Polish health system’ (P18, Hospital, Nurse, Poland)*

Transferred information should be structured, complete and timely. Some participants declared that nurses could be engaged to transfer the information between hospital and primary care. Between the hospital and LTC facilities social care worker is responsible for transferring the information. Informants consider the information transfer between these settings as optimal.

**Sub-theme 3: Availability & coordination of resources**

One of the most important, overarching factors that may affect care transition refers to the lack of regulations regarding care transitions, and therefore, their development should be a starting point. Moreover, participants expressed frustration with the limited availability of places in LTC and the limited availability of LTC staff to provide care and expressed the urgent need to address these problems to solve associated long waiting times to access LTC.

*‘For the patient to get to this long-term care (in-home long-term care provided by nurse), there is a waiting list, and this waiting list is sometimes several years, several months’ (P19, Primary care, Nurse, Poland)*

*‘Well, because if there is no free space, you can't move the patient, right? You can't discharge (from the hospital) because there's no place, and it's going to get worse and worse.’ (P23, Payer/Insurer, Management, Poland)*

Nonetheless, participants suggested also that the problem with the availability of the staff can also be found in different settings such as hospitals and social care institutions. Moreover, most participants suggested that a lack of coordination between the health and social sector and providers is a serious threat to care transitions. As a result, patients and/or caregivers may be forced to look for a nurse to provide care at home by themselves.

‘And what does he (caregiver) do? Looking for a nurse by word of mouth. Well, this is not how the system is supposed to work as a word-of-mouth method’ *(P19, Primary care, Nurse, Poland)*

To address the lack of coordination, most of the informants agreed that there is a need for a coordinator responsible for care transitions.

‘Someone should connect this care from the beginning to the end, that is, propose various possible variants, choose the most optimal model together with the patient and the family and bring it to the end’ *(P21, Long-term care, Management, Poland*)

On the other hand, some participants questioned the introduction of the care coordinator and its impact on the care transition. Moreover, there is a lack of agreement among participants on who should fulfil the role of care coordinator. Most of the participants suggested that the coordinator should be linked to the community and know the environment, providers, and the patient. As a result, some acknowledged the important role of primary care in care transitions. Nonetheless, according to a few participants, primary care settings are not fulfilling their role in monitoring and coordinating the patients and, thus, suggest stronger involvement of primary care. Contrary to that, some argued that primary care is too overburdened to be actively involved in coordination. At the same time, some informants emphasized the important role of nurses, care assistants, physiotherapists, charities, and volunteers. Particularly care assistants are found to be important actors for patients at home.

*‘We work a lot with the caregivers (care assistants). I can even say that my cooperation with care assistants is greater than with a doctor. Because the care assistant is simply with the patient all the time and if something happens to the patient and the patient cannot call, they call’ (P19, Primary care, Nurse, Poland)*

**Sub-theme 4: Training and education of staff**

According to participants, poor or even lack of education at the university regarding transitional care and the important role of nurses and their responsibilities could have an impact on care transition.

*‘Just as I think it is because they have nothing on this subject from academic education, and certainly such training (on transitional care) is very much needed’ (P19, Primary care, Nurse, Poland)*

*‘Why do we educate people at university ... and I am (the family nurse) supposed to clean the computer, take care of the paper in the printer, there are such tasks written on two whole pages and this is what students of family medicine learn’ (P19, Primary care, Nurse, Poland)*

Thus, strong emphasis on such education should be put. Besides, informants argued that there is a need for additional training for staff – healthcare staff, care coordinators (if available), care assistants etc. According to participants, nurses are not trained to provide the patient with information regarding care in other settings, and as a result, do not provide such information. Training could include providing education and information about good practices in transitional care and education regarding the organization of the LTC system.

*‘…having knowledge himself (the personnel), he may be more willing to undertake educational activities for the benefit of the patient and the family, right? He (the personnel) will not be afraid of questions from his (patient) family, because he will be prepared for them, so not only will he know himself, but it will translate into these benefits for the family because he will undertake these educational activities. If someone doesn't know, then he withdraws from it, because he doesn't know.’ (P21, Long-term care, Management, Poland)*

Participants argued that particularly face-to-face training instead of providing information leaflets would be more effective as staff could ask questions. Informants reported that currently, there are certain training options available not only for healthcare staff but also for the care assistants to provide care.

*‘Now there is a fashion for medical caregivers, so there are special trainings that are designed to take care of typical nursing patients, but not necessarily medical ones.’ (P17, Hospital, Social care worker, Poland)*

**Sub-theme 5: Education and involvement of the patient and/or caregiver**

Informants argued that patients and/or caregivers lack preparedness for discharge, which may lead to suboptimal care transitions.

*‘Either he (the patient) will hurt himself or someone will hurt him from the household, right, when he is poorly educated.’ (P19, Primary care, Nurse, Poland)*

According to participants, patients and their caregivers tend to be left on their own as there is no obligation from the personnel to provide information to the patient regarding care in other settings.

*‘Sometimes, unfortunately, the patient is discharged home […] and is somewhat on his own. If he doesn't ask himself or the family doesn't ask, then unfortunately there is such a search later ... a bit in the dark.’ (P18, Hospital, Nurse, Poland)*

On the other hand, if information is provided, it is short and unstructured. This results in patients’ and caregivers’ feeling lost and in need of informational and educational support.

*‘They (patients and caregivers) don't know where to start and what to do.’ (P21, Long-term care, Management, Poland)*

More than half of country informants agree that providing information and education to the patient and/or caregiver plays a crucial role as it affects patients’ and caregivers’ knowledge and involvement in care transitions. According to participants, education and information should be provided not only in the hospital but also at home and in primary care settings. Informants suggested that patients and/or caregivers should be provided with multidimensional information and education addressing all the patients’ and/or caregivers’ needs, including medical and non-medical. They further argued that some additional information and education could be provided with the use of leaflets, instructional videos or face-to-face training if needed.

**Sub-theme 6: Telemedicine and e-Health**

Telemedicine and e-Health are other important organizational aspects that may affect care transition in long-term care systems. Participants in their responses referred to the use of telemedicine and e-Health not only by health professionals but also by patients and/or caregivers. Participants suggested that the use of telemedicine and e-Health in Poland is rather limited. One informant argued that there is a lack of funding for telemedicine, and if there is funding, it is rather episodic. Nevertheless, more than half of the informants agreed that telemedicine and e-Health might be useful for remote patient monitoring of some patient groups. Moreover, according to participants, telephone consultations, and in general tele-information, are also useful tools to provide medical advice, referrals, and information to patients and their caregivers. Such tools enable the patients’ and their caregivers to access the information instantly and remotely. Video consultations were also acknowledged by the informants as a useful tool for communicating with the patient and their careers.

*‘…but it is also possible to use these visual systems, for example, from mobile phones and there to assess the patient's respiratory rate or check whether the device that has an automatic pressure measuring device puts it on correctly and measures the pressure well, these are I believe it actually makes a significant difference to improving care.’ (P20, Primary care, Physician, Poland)*

On the other hand, participants suggested that patients may avoid using telemedicine, or may lack knowledge on how to use it, and this may ultimately negatively impact their health. One informant argued that patients using telemedicine might be more neglected when compared with those using traditional medicine.

*‘…patients prefer to avoid…as if they can't get to a doctor in normal stationary conditions […] so this telemedicine has a big impact, because in fact they are a bit neglected, I would say, in the medical sense and also in the nursing sense, unfortunately.’ (P18, Hospital, Nurse, Poland)*

Two participants raised a subject on the use of online platforms to transfer the patient’s information between the providers, and in general, the importance of digitalization.

**Sub-theme 7: Social care**

More than half of country informants argued that social care institutions and social care workers, with their proactive engagement and involvement, play a very important role in care transitions. Participants highlighted that social care workers help not only to prepare the documents prior to discharge and to communicate with the LTC providers but also help to arrange the place of care for the patient in the next setting.

*‘…social worker, workers…and they deal with the role of looking for a place in a specific center and institution where the patient needs. If it is a discharge home…then also help the family a bit and provide them with information on where they can look for help even later in the living environment.’ (P17, Hospital, Social care worker, Poland)*

Thus, participants expressed the need to proactively engage social care workers to provide holistic care and support for older adults and their caregivers not only in the hospital setting but also at home. For patients at home, social care workers could be involved in supporting patients’ daily functioning by delivering hot meals, socializing or patient monitoring. According to an informant, such involvement may prevent unnecessary healthcare utilization. Nonetheless, some participants agreed that the number of social care workers is insufficient to address the patients’ and caregivers’ needs. Moreover, informants argued that social care workers often could not actively be involved in patient cases due to their tasks being limited to administrative activities and their limited competencies.

*‘There are social workers who should be dealing with such a transition of these patients. On the other hand, they... I guess there are too... there are certainly not enough of them and the scope of their competences, the scope of their activities is very limited.’ (P22, Long-term care, Management, Poland)*

*‘The role of a social worker is practically limited to administrative activities. Unfortunately, this is bureaucracy, and it's cosmic.’ (P17, Hospital, Social care worker, Poland)*

Additionally, some patients and caregivers do not know where to access help from social care institutions, as stated by the participant. Besides that, one informant suggested that there are some differences in the involvement of social care settings/workers, with some being unresponsive, disorganized, and always late.

*‘There are social welfare facilities that operate very dynamically, and this patient is taken care of, and there are those that need to be pressured…to take care of (the patient).’ (P21, Long-term care, Management, Poland)*

**Sub-theme 8: Supporting informal caregivers**

More than half of the informants in Poland agreed that the support provided to the informal caregivers in the settings is very limited or even non-existent, and thus, patients and their caregivers often need to search for support and help on their own. One informant suggested that even if such support is provided, it is often short and unstructured. According to participants, limited access to support may be the result of a lack of formal requirements for the providers to provide support to informal caregivers.

*‘If there is a good, kind, e.g., ward nurse, she will tell you at discharge, she will also instruct you…but in the system, well, it doesn't work. This is someone's goodwill, but it is not so obligatory that if there is a discharge card, there must be a workshop with the family, a meeting and a thorough discussion.’ (P21, Long-term care, Management, Poland)*

Half of the informants agreed that informal caregivers should be provided with educational, information and instrumental support if needed. They also realize the need for a coordinator that could guide the patient and the carer throughout the entire process by providing medical, administrative, and legal support. Moreover, according to informants, the availability of training for informal caregivers and limited or even no access to respite care may also have an impact on the care transition of older adults.

**Financial challenges**

The knowledge of some participants regarding financial aspects was limited and for this reason, some informants were unfamiliar with the impact of reimbursement, rewards and penalties on care transition. Nevertheless, informants in Poland agreed that reimbursement-related factors particularly have an immense impact on the care transition of older adults.

**Sub-theme 9: Reimbursement**

The role of reimbursement and its impact on care transition was one of the most discussed subjects among Polish participants. According to more than half of the informants, out-of-pocket payments are one of the most important factors affecting care transitions by restricting patients and their caregivers from accessing LTC services.

*‘…if it is financing the so-called commercial, then the patient simply cannot afford it and stays at home’ (P19, Primary care, Nurse, Poland)*

*‘…we also observe the phenomenon that these families simply leave 70 percent of these benefits (patients’ pension) for everyday functioning. They can't afford to donate 70 percent.’ (P21, Long-term care, Management, Poland)*

Apart from that, some of the informants agreed that underestimated contracts, low LTC provider reimbursements, and low salaries for LTC staff might affect the availability of LTC places and services, and, thus care transitions. Additionally, participants suggested that fixed contracts with LTC facilities and a low number of contracts for LTC staff are other problems that need to be addressed. As a result, informants suggested the need for better estimation of the contracts for LTC facilities, increasing salaries and the number of contracts of LTC staff. According to participants - charities, volunteers, non-governmental organizations, and the European Union play an important role by providing financial support to LTC facilities and staff. Informants also discussed different ways of paying providers together with their advantages and disadvantages in relation to care transition. For example, some argued that budgets could be used to pay for transitional care, while others stated that family nurses should receive additional reimbursement besides per capita payment for additional services provided. Moreover, participants also argued that the use of fee-for-service payments and, in general, activity-based payments might negatively affect care transitions. On the other hand, they spoke in favor of introduction of quality-based reimbursements while at the same time acknowledging the difficulties in measuring quality of care.

*‘It's not so simple to measure quality.’ (P23, Payer/Insurer, Management, Poland)*

A few informants mentioned that in Poland there is no separate reimbursement for transitional care and that the introduction of ‘satisfactory’ reimbursement for the coordination/coordinator/transitional care could lead to optimization of care transitions. One participant stated that current reimbursement for care coordination in primary care is not satisfactory and does not motivate them to provide coordinated care. Moreover, some informants argued that there is a lack of stable governmental funding for telemedicine, training for caregivers, and care homes and that creating LTC wards next to the hospital may improve patient flow and decrease the total costs.

**Sub-theme 10: Rewards**

According to Polish informants, there are no rewards for providers in the Polish health and social system. Nonetheless, some participants argue that there is a need for the introduction of rewards as they may positively affect care transitions. Few informants suggested that not only the care coordinator, if available, should be eligible for rewards but also providers whose additional activities lead to the improvement of quality of care. On the other hand, some participants raised an issue of measuring quality of care, and thus, appointing those eligible for the reward. Additionally, one participant argued that healthcare professionals should not be motivated by rewards, nor penalties due to the nature of their work.

*“…and counting on a reward or a punishment - I think that in our professions, it's probably ... It's not an industrial plant that I will produce something more and then I will get a bonus for it or I will get a penalty because I did not complete the contract.’ (P19, Primary care, Nurse, Poland)*

**Sub-theme 11: Penalties**

The subject of penalties has polarized the country informants. Some participants recognized the potential of using penalties, while others questioned their implementation. Nevertheless, more than half of the informants suggested that penalties could improve care transitions if they were issued for inappropriate care, adverse events, different kind of abuse, misuse, and abnormalities or for not fulfilling the contract. Moreover, one participant argued that penalties could also be implemented for missing information in the referrals and for unnecessary referrals. Others suggested that penalties for providers could be harmful and, for instance, burden already strained budgets.

*‘And on the one hand…there is a system of penalties and rewards, but on the other hand, it also makes it necessary for medical entities to pay back these penalties, makes them financially burdened and does not fully secure them. They have a problem with securing their basic needs.’ (P22, Long-term care, Management, Poland)*

For this reason, one participant suggested that penalties are sometimes inadequate and should be rather symbolic and constructive. Another dilemma regarding the penalties referred to estimating the responsibility if something goes wrong and difficulty in measuring quality of care. Some participants stated that currently, there are no penalties for suboptimal care transitions. However, if there were care coordinators, such penalties could be introduced.

**Appendix 6. Basic findings from the interviews**

| **GERMANY** | |
| --- | --- |
| **Organizational aspect** | **Basic findings from the interviews** |
| Communication | - Availability of round-table regular meetings with different professionals from other settings and sectors (P06, P05, P01) - Need for better communication between different professionals and sectors involved in care process (P03, P07, P02) - Knowing personally involved professionals/institutions ease communication (P01, P04) - Need for more communication about patients’ needs (P04, P08) - Need for personal communication with patients and family (P04, P08) - Importance of communication of 3 sides (sending-patient-receiving) (P01) - Receiving and sending settings should communicate for planned transitions of vulnerable patients (P03) - Importance of verbal communication (P03) - Importance of electronic or digital ways of communication (P03) - Need for the central actor facilitating the communication about free places in long-term care (P01) - Need for on-time communication (P08) - Communication between social care worker (sozialdienst) and LTC facilities (P06) - Communication between ambulatory and stationary LTC regarding patient health status (P06) - Good communication with the primary care (P05) - Need for more detailed communication (P05) - Limited communication between involved professionals (P04) |
| Limitations in communication | - Limited communication with the family (P01, P05) - Using outdated methods (on paper) to communicate with other providers (P02) - Communication with the hospitals is malfunctioning (P05) - Limited involvement of patient and family (P05) - Involved groups may not understand the information (official language/formal language) (P04) |
| Transfer of information and patient responsibility | - Need for electronic health information exchange, specifically electronic patient record (P03, P02, P06, P04) - Importance of standardized protocol for information exchange (P02, P06) - Need for more detailed information (P08, P05) - Importance of e-Health (P03) - Some improvements were observed in some places in Germany, health care providers worked to develop standardized transition protocol (P02) - In complex situations doctor telephone, the general practitioner for clarification (P01) - Transfer of information should be going through the insurer (Plegekasse) (P07) - Patients’ preferences should be also included (P08) - Need for more enhanced transfer of information (P05) - Transfer of information is performed by social care worker (discharge manager) or by ambulatory care provider (P06) - Need for timely information on disability score or the patient and organization responsible for financing (P06) - Good transfer of information from primary care (P05) - Round-table meetings to exchange ideas and information (P05) - During transition from home to LTC – patient’s family is responsible for providing information (P06) |
| Limitations in transfer of information and patient responsibility | - Receiving non-specific, incomplete, delayed, or even no information is delivered (P02, P01, P05, P04) - Using outdated methods such as on paper or fax to transfer the information (P01, P02, P04) - Transfer of information may be affected by data protection (P08, P04, P02) - Need for improving transfer of information (P01, P05) - Standardized protocol is not used in routine care (P02) - Very limited use of electronic patient records (P02) - Involved groups may not understand the information (official language) (P04) - Transfer of information is the worst with primary care providers (P06) |
| Coordination of resources | - Need for better interprofessional and intersectoral collaboration among all involved in care process (P03, P01, P07, P08, P05) - Need for clear definition of responsibilities of professionals and organizations (P01, P02, P08) - Need for patients’ assessments, getting to know them personally & their needs (P01, P02, P08) - Importance and need for case management/manager (P02, P01, P08) - Case managers role should be to focus more on patients’ and optimization of care and to look beyond the current setting (P02) - Case manages could also take on social aspects (P02) - Advanced practices nurses could be case managers (P02) - Need for care planning and transition planning (P02, P08) - Importance of LTC infrastructure (staff etc.) (P02, P08) - Regular meetings with involved professionals and institutions (P06, P05) - Legal regulations regarding discharge planning in hospitals and disability scale (P07) - Need for losing restrictions and reducing bureaucracy regarding hiring nurses from abroad (P06) - Organizations should be aware of work of other settings so that the patient is prepared prior to care transition (P03) - System should be designed to enable professionals to adapt procedures and activities to the needs of the patients, if possible (P03) - Adapting structures and routines for intersectoral and interprofessional collaboration (P03) - Important positive role of the payer in planning care (P01) - Visits of medical staff at the LTC facilities to avoid hospitalization (P01) - Need for the support from the management (P02) - Importance of implementing transitions of care models by the organization (P02) - Importance of having case conferences (P02) - Need for more staff with one year training, less specialized (P07) - Discharge and transition planning should be performed together with payer (P07) - Providing essential medicine during discharge over the weekend (P06) - During transition from home to LTC – patient’s family is responsible for providing information (P06) - Need for higher involvement of care assistants during transition process (P06) - Need for higher involvement of primary care (P06) - Availability of places to support patients & informal caregivers (P06) - Doctors have vital role in selecting patients’ transition destination (P04) - Doctors have vital role in selecting services provided to the patient in ambulatory care (P04)   Longer established providers are more preferred than newcomers (P04) |
| Limitations in coordination | - Limited availability of staff especially in LTC (P01, P07, P06, P05) - Limited availability of places in LTC facilities (P01, P06, P05) - Lack of coordination between health and long-term care providers (P04) - Normative and legal conditions may make it difficult to delegate or transfer responsibilities (P03) - Organizations implement their own protocols but these protocols are not adapted to the situation of the patients (P03) - Separate financing streams for health care and long-term care (P01) - Using weekends and nights by LTC facilities to transfer “difficult” patients to the hospital (P01) - No care planning/transition planning means more suboptimal care transitions (P02) - It is common in Germany that unclear responsibilities of the professionals lead to suboptimal transitions (P02) - Case managers in Germany are focused on optimization for hospitals instead of optimization of care for the patient (P02) - Limited use of case conferences (P02) - Lack of legal regulations for carers from abroad (P07) - Doctors have dominant role (P07) - Nurses are not independent in their decisions (P07) - Competing for the budget between medical doctors and other providers (P07) - Limitation in division of care of staff with more training and less training (P07) - Lack of planning regarding the location of LTC facilities (P07) - Overproviding of ambulatory intensive LTC care (P07) - Limited involvement of primary care (P06) - No formal referrals for LTC (P06) - Primary care physicians are overworked (P06) |
| Education and involvement | - Patients’ and caregivers’ needs and preferences should be considered, care should be patient-centered (P03, P02, P07, P08) - Importance of providing information and education to the patient and caregivers (P03, P02, P08, P06) - Need for involvement of patients and caregivers in decision-making process (P05, P01, P08) - Importance of involving caregivers in the care process (P03, P01, P08) - Providing information and education to the patient and caregivers (P01, P06, P04) - Availability of places providing advice and information (P07, P06) - Regular meetings with the patients and family (P01) |
| Limitations in education and involvement | - Patients’ and caregivers’ needs and preferences are not considered (P03, P02, P05, P04) - Limited involvement of patients’ and caregivers’ in decision-making (P03, P05, P01, P02) - Informal caregivers are often poorly informed and involved (P02) - Limited use of available advice, help centers by patients and caregivers (P06) - Decisions of patients regarding the selection of care activities may be inappropriate (P05) |
| Training and education of staff | - Need for well trained staff (e.g. case managers, LTC staff, care assistants) to provide high quality care (P08, P06, P03, P07) - Staff should develop competencies to look at care from multiple perspectives, also perspective of other providers (P03) - Professionals should have basic knowledge about how the care is organized in other settings (P03, P07) - Availability of well trained staff in the hospital (P06, P01) - Mandatory training courses for the staff (P06, P05) - Change in training scheme for nurses, comprehensive training programme (improved competencies) (P07) - More attention should be paid to the training regarding the communication/transfer of information (P01) - Need for increasing awareness of the staff regarding care transition (P02) - 1-year training for care assistants to provide all non-medical services (P07) - Employers compete with each other in order to keep personnel by providing additional trainings to the staff (P05) |
| Limitations in training and education of staff | - Staff may not be competent enough to assess patients’ needs (P03) - Nurses and care assistants are not trained to perform activities independently (P07) - In the past, division within training scheme among nurses (specialized to provide care only for some specific age groups) (P07) - Lack of psychological help/assistance provided to the staff. Need for more (P05) - Lack of training regarding transitional care (P04) |
| Telemedicine and e-Health | - Need for higher use of telemedicine and e-Health (P02, P03, P08, P06, P05, P06) - Need for electronic patient record (P06, P03, P02, P08) - e-Health is a mediator but not a causal factor for patient-centered care (P03) - Importance of video consultations (P02) - Importance of telemedicine, tele-nursing etc. (P02) - Introducing video-consultation (P07) - Use of health monitoring devices (P06) - Need for use of health monitoring devices (P05) |
| Limitations in telemedicine and e-Health | - Very limited use of telemedicine and e-Health (P01, P02, P04, P06, P05, P06, P03) - Use among older adults can be problematic (P01, P02) - Very limited use of electronic patient records (P02) - Limited use of telemedicine affects possibility of patients to be discharged to home (P02) - Skeptical attitude of primary care towards video consultations (P07) |
| Social care | - Social care institutions are responsible for covering the costs for LTC if patient and/or family are not capable to pay (P01, P06, P07) - Social care institutions help patient/caregiver to cover the costs of LTC without issues (P06, P01) - In inpatient setting, social care workers are focused on discharge management, for example by preparing the receiving setting (P03, P06) - Importance of involving social care workers in the interprofessional/intersectoral meetings (P02, P07) - The importance of good functioning discharge manager (Sozialdienst) in the hospital (P08) - Availability of social services in nursing home and hospitals (P02) - Involvement of social care worker (discharge manager) in hospital, regular meetings with providers and working on problem-solving (P06) - Social care institutions are interested in lowering rates for LTC facilities (P07) |
| Limitations in social care | - Participant have mixed feelings regarding involvement of social care workers in discharge planning (P03, P02) - The role of social care workers is limited in outpatient settings (P03) - Difficulties in communication with social care institutions (P05) - Unwillingness to pay by social care institutions to support LTC placement in case of cost rise (P05) - Social care institutions communicate with patients and/or families in incomprehensible way (P04) - Having separate role of social care worker in the hospital may lead to diffusion of responsibilities (P02) |
| Supporting informal caregivers | - Availability of training courses for informal caregivers (P01, P02, P06, P05, P04, P08) - Availability of respite care services (P06, P07, P08) - Patients receive cash benefits to pay to carer of their choice. Thus, informal caregiver receives financial compensation (P07, P08, P01) - Availability of centers providing information and help to informal caregivers (P06, P07) - Need for increasing the budgets for respite care (P06) - Importance of assessing informal caregivers’ needs (P02) - Informal caregivers should also receive a training on how to take care of themselves (P02) - Importance to provide information about the institutions and professionals that provide help or respite care (P02) - LTC staff helps to identify patients at home that should be transitioned to formal LTC facilities (P04) - Payer contributes to pension insurance of informal caregivers. The level depends on disability score (P07) |
| Limitations in supporting informal caregivers | - Sometimes offered courses and information centers are not used by informal caregivers (P06, P04) - Informal caregivers do not receive enough support during care transition, are not involved & informed sufficiently (P03, P02) - Informal caregivers do not receive ongoing support/education/information (P02) - Need for more solutions regarding respite care (P07) - Monetary compensation for respite care is prone to fraud from the applicant’s side (P07) - Informal caregivers don’t always receive sufficient support, it depends on family’s determination (P08) - Limited psychological support provided to informal caregivers (P08) |
| **Financial challenges** | **Basic findings from the interviews** |
| Reimbursement | - Social care support for patients’ in reimbursement for LTC facilities in case of lack of financial funds/property/inability to pay by families (P06, P05, P07, P01) - Profitable reimbursement rates for LTC facilities & fixed and in general satisfactory salaries for staff (P07, P01) - Participants’ mixed feelings regarding value-based reimbursements (P03, P02) - Reimbursement in ambulatory LTC is satisfactory (P05, P04) - Availability of LTC insurance (P01) - Importance of evidence-based reimbursements – reimbursing what works (P03) - Two possible reimbursements for psychiatric hospital for LTC patients: DRG or per-diem (P01) - Financing should be in one hand and the same across the sectors to reduce barriers between the settings (P02) - Reimbursing video consultations (P07) - Reimbursing trainings for informal caregivers (P07) - Negotiating reimbursement rates with social care and providers (P07) - DRG reimbursement in hospitals + budget (P07) - Per-diem reimbursement dependent on disability score (P06) - The need for higher salaries for ambulatory LTC staff instead of additional rewards (P05) - Need for higher public funding for LTC (and reducing OOP) (P04) - Need for additional lump sum payment for transition period before the patients’ disability score is estimated (P06) |
| Limitations in reimbursement | - The role of out-of-pocket payments for LTC (P07, P06, P05, P04, P08, P01) - Lack of reimbursement for interprofessional collaboration/intersectoral care/transitional care (P03, P02) - Payments per-diem may have negative impact on quality-of-care or admissions and ultimately care transitions (P07, P08) - Activity-based payments may have negative impact, for instance, on supplier-induced demand (P02, P07) - In Germany, reimbursement is physician-centered, focus on physician needs (P03) - DRG reimbursement in hospital may shorten the length-of-stay without justified cause (P06) - Value-based payment methods – difficulty in measuring quality (P02) - Payments per-diem are not flexible enough. Need for additional lump sum to compensate for variability in incurred costs (P06) - Extensive administrative work related to the reimbursement and reporting (P01) - Reimbursement of services may be restricted to some age groups (P01) - The level of reimbursement is dependent on the score on disability scale. Sometimes the disability score is provided with the delay (P06) - The level of reimbursement is dependent on the score on disability scale. Responsible institutions often manipulate the score for their gain (reducing costs) (P05) - Unwillingness to pay by social care institutions to support LTC placement (P05) - Reimbursement for some ambulatory LTC services don’t correspond to the needed workload (P05) |
| Penalties | - Penalties are not available in Germany (P06, P05, P07) - Participant mixed feelings/hesitancy about the use of penalties (P03, P06) - Too minor penalties may not have desired effect (P03) - Penalties on their own are not sufficient measure, they need to be constructive and offer solutions (P02) - Penalties could help to raise awareness about the problem (P02) - No information provided by the respondent (P08) - Penalties could be enacted for misuse, abuse, abnormalities (P07) |
| Limitations in penalties | - Problems with appointing responsible party (P06, P07) - Difficulty in measuring the quality (P02) |
| Rewards | - Rewards are not available in Germany (P06, P01, P07) - Limited knowledge regarding rewards (P02, P03, P01) - Participants’ mixed feelings/hesitancy about the use of rewards (P06, P03) - Some potential for rewards to improve care transitions, stimulate practices (though participants unsure how) (P02, P03) - Additional payment during corona (P05) - Need for higher salaries instead of rewards (P05) - Importance of creating quality indicators related to care transitions (P03) - No information provided by the respondent (P08) |
| Limitations in rewards | - Problems with appointing responsible party (P06, P01) - Questioning whether rewards are effective in long-term (P03) - Difficulty in measuring the quality (P02) - Problems with ‘cheating’ the system by ‘pretending’ that criteria are met (P02) |

PXX –ID of a participant (as shown in Table 2: Participants’ characteristics)

| **THE NETHERLANDS** | |
| --- | --- |
| **Organizational aspect** | **Basic findings from the interviews** |
| Communication | - Importance of good communication with the patient and informal caregiver (P16, P11, P15, P13,14, P21) - Importance of interprofessional/intersectoral communication (P09, P10, P16, P15, P21) - Availability and importance of multidisciplinary team meetings for interprofessional/intersectoral communication (P09, P15) - Importance of e-Health solutions to improve communication between the providers and also with the caregiver (P09, P16) - Important role of transfer nurses communicating with receiving setting (e.g. community care, LTC institutions) and informal caregiver (P09, P16) - Important role of social care workers communicating with LTC institutions, the patient and informal caregiver (P09, P15) - Good communication between providers, especially home care and long-term care (P11) - Professionals themselves contact the long-term care facility (P11) - Professionals from long-term care facilities contact the person in home care to get acquainted (P11) - Knowing professionals from the organization/institutions ease the communication (P09) - For patients being discharged home without need of care, general practitioner or community physician is contacted (P09) - Importance of video call or telephone call to communicate with other providers (P10) - Institutions that are part of the hospital communicate easier and better (P16) - Importance of good communication between the providers about patients’ medical, psychological, social and caring needs (P16) - Important role of client advisors in communicating with institutions, patient and informal caregiver (P13,14) - District nurses communicate with the LTC institutions P13,14) - District nurses may also visit and communicate in person with the staff at the LTC institutions (P13,14) - Involvement of the district nurse in communication between the professionals, the patient and informal caregiver (P13,14) |
| Limitations in communication | - Sometimes interprofessional/intersectoral communication is not optimal, particularly between hospitals and home care (P10, P16) - Need for improvement of communication (P16) |
| Transfer of information and patient responsibility | - Importance of good transfer of information between the providers/institutions and informal caregivers (P09, P10, P15, P12) - Importance of the quality of transferred information, for instance, completeness (P11, P10, P15, P13,14) - Availability of agreements between the providers may improve transfer of information (P09, P16) - Importance of good transfer of patients’ information including not only medical but also psychological and social aspects (P15, P11) - Importance of standardized protocol for information exchange (P09, P15) - Important role and need for electronic health records (P16, P15) - Importance of timely transfer of information (P10) - Need for transferring information to all providers involved in the next setting also including patients’ preferences (P10) - If patient goes to the hospice, Information is provided with the letter and follow-up call to the GP (P15) - Importance of telephone call while transferring the information (P15) - Interprofessional collaboration may smooth the transfer of information (P15) - All providers should have access to agreements concerning advance care planning (P15) - In some cases, meeting in-person with the staff at the receiving setting may improve transfer of information (P13,14) - Good transfer of information between providers (P13,14) - Information about the patient is available in the medical file, records (P13,14) - Information about the patient is passed with the use of secured mail (P13,14) |
| Limitations in transfer of information and patient responsibility | - Sometimes transferred information is not detailed enough, is incomplete (P09, P10, P15) - Sometimes transferred information is delayed (P09, P10, P15) - Privacy laws may restrict transferring the information between the institutions (P09, P16) - Lack of single system for information exchange, every provider has their own system (P15, P12) - Transfer of information is one of the biggest flaws in the Netherlands (P09) - Sometimes transferred information includes only one providers’ perspective (P09) - Lack of participation of the provider in digital solutions to transfer information (P09) - Time pressure to transfer the patient to another setting (P16) - The information about the psychological aspect is often not transferred (P15) - Diminished responsibility of who should transfer the information (P15) - Provision of wrong information may affect care transition (P13,14) |
| Availability and coordination of resources | - Importance and need for better interprofessional/intersectoral collaboration among all involved in care process (P11, P09, P10, P15, P12) - Availability and importance of agreements between the providers/institutions (P09, P15, P16, P13,14) - Importance of LTC infrastructure (e.g. sufficient number of staff, need for more institutions, availability of crisis beds in the nursing homes) (P09, P16, P13,14) - Important role of physiotherapist (P09, P10, P16) - Importance of good patients’ assessment and indication (P09, P10, P12) - Availability and importance of multidisciplinary team meetings (P09, P15) - Need for clear definition of responsibilities of professionals and organizations (P11, P12) - Primary aim is to keep the patients as long as possible at home (P13,14, P12) - Availability and important role of transfer nurses (P09, P16) - Need for the awareness and support from the management regarding transitional care/collaboration (P09, P12) - Importance of advanced care planning/transition planning and access to such plans by all providers (P09, P15) - Importance of engaging community nurses during care transitions (P10, P13,14) - Importance of care transition managers in the long-term care settings (P16) - Need for involvement of professionals from previous setting in the next setting (e.g. long-term care facility) (P11) - Importance of timely involvement of different professionals and timely follow-up (P10) - Importance of application of transitional care interventions in regular care (P10) - Need for integrating transitional care interventions in already existing care networks, not building new ones (P10) - Primary care physician and nurse could have an important role within transitional care interventions (P10) - Transitional care interventions should consider home as a starting point so that transitions are prevented in the first place (P10) - Important role of dietician (P16) - Knowing personally involved professionals/institutions (P15) - In urgent cases, care in nursing home is organized within few days (P13,14) - Important role of client advisors in preparing receiving setting and the client (transition from home to long-term care institutions) (P13,14) - Importance of understanding the interrelation between the reforms and impact on different actors (P12) - Reforms should be considering the impact on the whole system, not only on single organization (P12) |
| Limitations in availability and coordination of resources | - Limited availability of staff (P09, P12) - Criteria for obtaining Wlz (indication to receive LTC care) is strict, not rational (P09, P13,14) - Lack of collaboration between providers/institutions - working in silos (P09, P12) - In some cases, waiting time, waiting list to access the next setting (P15, P13,14) - Waiting time for the indication Wlz that enables the patient to access long-term care home or other care institution (P13,14) - Fragmentation within the organization when it comes to responsibilities (P11) - The size of the organization may impact the care transitions, the bigger the organization, the more difficult care transitions (P11) - Patient moving from location A (e.g. home) to location B (e.g. nursing home) receives new physician, nurse, medication system (P11) - New case manager introduced as a part of transitional care intervention may be unfamiliar with the patient and his/her network (P10) - Certain rules and regulations may affect care transitions (P16) - Reforms in one part of the system may have an unintended consequence for other involved actors (P12) |
| Training and education of staff | - Staff should be aware or trained about work of different professionals, in other settings (P09, P10, P13,14) - Importance of education regarding transitional care (P16, P15) - Important role of multidisciplinary team meetings in getting to know about each other’s work (P09, P10) - Staff is well trained and educated (P11) - Importance of educating the staff to recognize some disease specific vital signs (P10) - Importance of providing additional training to staff to improve quality of care transitions (P16) - Importance of training and education of staff to provide the right information to the other professionals (P13,14) - Importance of changing the mindset of professionals from “taking over” care from the patient & informal caregiver to more interaction & support-based model (P12) - Educating staff about the important role of providing support for self-management (P12) |
| Limitations in training and education of staff | - Staff having limited knowledge about work of other professionals, other settings (P09) - Staff in the community setting has rather generic geriatric education and may have difficulty in dealing with complex patients with specific diseases (P10) |
| Education and involvement | - Importance of well-educated and informed patient and informal caregiver (P09, P16, P10, P15, P13,14, P12) - Importance of providing multidimensional information/education to the patient and informal caregiver (P09, P10, P16, P15) - Importance of patients’ and informal caregivers’ needs and preferences (P09, P10, P15, P12) - Importance of the involvement of informal caregivers in the care process (P10, P12) - Importance of assessing caregivers’ ability to provide care (P10, P12) - Assessing informal caregivers’ ability to provide care (P15, P12) - Providing multidimensional information/education to the patient and informal caregiver (P13,14, P12) - Providing the patient and informal caregiver with information at an early stage (P13,14, P12) - Need for engagement of informal caregiver in long-term care facility (P11) - Importance of providing the patient and informal caregiver with information and education at an early stage (P09, P10) - Need for addressing psychological needs of the patient and informal caregiver (P16) - Professionals knowing personally involved professionals/institutions can provide more detailed information (P15) - Involvement of patient and informal caregiver in decision-making process (P15) - Providing education and support to the patient and informal caregiver for self-management (P12) |
| Limitations in education and involvement | - In some cases, informal caregivers are not involved in the care process, or their involvement is very limited (P09, P10, P11, P12) - Involvement of the informal caregiver is very limited once the patient is transferred to long-term care facility (P11) - The level of involvement of the informal caregivers depends on the organization (P09) - Sometimes provided care is not patient-centered (P16) - Lack of information and education to the patient may prolong recovery (P16) - Some informal caregivers may be afraid/hesitant to ask questions (P16) - The education and information provided to the patient and informal caregiver varies among providers/institutions (P16) |
| Telemedicine and e-Health | - Important role and the use of electronic devices to monitor patients at home (P10, P16, P13,14, P12) - Important role and the need for electronic patient record that is accessible to all (P09, P10, P16) - Important role of e-Health and telemedicine to provide optimized care transitions (P12, P10) - Need for telemedicine and e-Health solutions to be personalized (P15, P09) - Use of telemedicine at home, for instance medication dispenser (P11) - The use of telemedicine is helpful in self-management (P11) - e-Health could improve standardization (P09) - Importance of providing e-Health, telemedicine resources to the patient (P10) - Importance of providing e-Health and telemedicine resources to the staff (P10) - Telemedicine/e-Health devices may improve communication with the family (P16) - Participants mixed feelings regarding the use of telemedicine and its effectiveness (P15) - Need for more testing of e-health and telemedicine solutions (P12) |
| Limitations in telemedicine and e-Health | - The use of telemedicine stops once the patient is transferred to long-term care facility (P11) - Lack of integration of the provider in digital solutions (P09) - Privacy issues (P10) - Patients’ inability to pay for electronic solutions, telemedicine (P10) - The use of telemedicine among older adults is not very common (P15) - Older adult patients have complex needs that may not be addressed with tele-health (P15) - Future generations will be more digital competent due to current use of digital solutions (P12) |
| Social care | - Availability and important role of social care workers in hospitals and home care (P11, P09, CS, P15) - Important role and involvement of social care workers in preparing the transition, for instance, to nursing home (P11, P09, CS, P15) - Social care workers have more time to look at other aspects beyond medical care (P09) - Important role of social care workers to assess the patients’ situation holistically (P09) - Social care workers are not always involved in care transition (P16) - Social care worker often knows the patient very well and their environment, needs and preferences (CS) - In some cases, social care worker can be seen as coordinator between different institutions (CS) - Important role of social workers in providing support to informal caregivers (P15) - Social care workers are well informed about different organizations providing care (P15) - Participant mixed feelings regarding the role of social workers (P13,14) - Social care workers can arrange volunteers (P13,14) - Social care workers may be engaged to provide support and assistance to the patient & informal caregiver, especially with non-medical tasks (P12) - Social care workers could socially engage the patients this could result in patients’ being more active and independent (P12) |
| Limitations in social care | - The role of social care worker could be performed by other professionals i.e. nurse (P09, P11) - Social components are not addressed enough (P11) - Social care workers know patients less than a district nurse (P13,14) |
| Supporting informal caregivers | - Support provided to the informal caregivers varies among organizations/institutions (P09, P16) - Participant believes that provided support is sufficient (P13,14, P12) - Providing informal caregivers with information, guidance, and support and/or bringing them in contact with right professionals (P13,14, P12) - Importance and need for psychological or social support provided to the informal caregiver (P10, P11) - Need for assessing informal caregivers’ needs (P11) - Participant doesn’t have firm opinion whether provided support to is sufficient (P15) - Important role of social workers in providing support to informal caregivers (P15) - Availability of organizations providing support to informal caregivers (P12) |
| Limitations in supporting informal caregivers | - Informal caregivers do not receive enough support during care transition (P11, P09) - Lack of structural involvement of informal caregivers in most settings (P09) - Some informal caregivers may be afraid/hesitant to ask questions (P16) |
| **Financial challenges** | **Basic findings from the interviews** |
| Reimbursement | - Need for reimbursing interprofessional/intersectoral collaboration, transitional care (P11, P10) - Importance of satisfactory salaries for the staff (P16, P17) - Participants mixed feelings regarding the activity-based payments (P10, P17) - The organization receives the budget, nurses receive salary independent of volume of care provided (P17, P10) - Participants limited knowledge regarding the reimbursement (P15, P13,14) - Importance of sufficient reimbursement level of providers/institutions (P17) - Participant mixed feelings regarding value-based reimbursements (P11) - Availability of extra quality reimbursement as a part of standard reimbursement, the effect is still unclear (P16) - Importance of value-based payments and their potential to improve quality of care (P17) - The government tries to keep the patients at home for as long as it is possible because it is cheaper than institutionalization (P11) - Need for flexibility to combine reimbursement forms from the government and health insurers (P11) - Need for increasing financing for long-term care (P17) - Reimbursement per patient should be based on what is declared by the caregiver what’s needed for the patient at given moment (P17) - Nurses themselves should do indication about patients’ caring needs (P17) - Community nurses are financed from basic insurance (P10) - Need for the reimbursement of physical therapy (P10) - Five years ago Dutch government gave a lot of extra money to improve the quality of care (P16) - Increasing salaries for nurses and careers increased the total number of the staff, it has an impact on time spent with patients (P16) - Health insurance company dedicate additional reimbursement for training staff to improve quality of care (P16) - Reimbursing long-term care organizations in advance results in possibility of organizations to secure beds, staff etc. (P16) |
| Limitations in reimbursement | - The role of out-of-pocket payments for LTC (P16, P15, P13,14, P12) - The reimbursement per patient is fixed according to indication, irrespective of variability in care needs, as a result some organizations may experience financial loss (P17, P10, P13,14) - Lack of reimbursement for interprofessional/intersectoral collaboration, for instance, when nurse from long-term care facility visit patient at home (P11, P10) - Value-based payments - Difficulty in measuring quality and keeping track of the entire transition process, need for standardized indicators (P11, P17) - Activity-based payments could have negative impact on care transition by, for instance, leading to overproduction (P11, P15) - Lack of flexibility/possibility to combine reimbursement forms from the government and health insurers (P11) - Low salaries for LTC staff, particularly community nurses (P17) - Physiotherapy is not included in basic insurance (P10) - Financial resources for long-term care is limited/low, this has implication on availability of beds in LTC (P17) - Insufficient reimbursement for providers/institutions may affect availability/staffing levels (P17) - The village or the city may be reluctant to pay for home care and may prefer moving the patients to long-term care, home care is paid by the city while long-term care is covered from tax (P16) - Reimbursement for extra nurses, social care workers etc. may not be paid by insurance companies or the government even if it is meant to improve quality of care (P16) - Some long-term care facilities complain about lack of financial resources to improve quality of care (P16) - Not flexible reimbursement arrangements, example of earlier hospital discharge and providing care at home (P16) - Cutting the budget (reform in 2015) in the Netherlands for long-term care had an impact on number of LTC settings (P12) - Cutting the budget for LTC organizations by the government – cuts will need to be done somewhere within the organization (P12) |
| Rewards | - Importance of internal motivations of staff to provide good quality care is more important than financial rewards (P11, P16, P15, P13,14) - Participant mixed feelings regarding the use of financial rewards (P11, P16, P12) - Financial rewards at the organizational level could improve quality of care by, for instance, encouraging collaboration between professionals/providers/sectors (P17, P16) - Participants negative feelings towards the use of financial rewards (P13,14) - Rewarding organizations once a year based on their performance (P17) - Financial rewards could be potentially reinvested by the organizations to further improve quality of care (i.e. staff, beds, education) (P17) - Rewards should be for organizations, not for individuals (P17) - System of rewards was used in the Netherlands (P16) - Stimulation is more effective than rewards. Money should be put into developing competencies of staff so that the effect is maintained (P16) - Financial rewards have potential to impact care transition, but on organizational level, not at healthcare professional level (P15) - Lack of financial rewards for district nurses (P13,14) |
| Limitations in rewards | - Financial rewards are short term stimulation (P12, P16) - Having rewards for long-term is not possible as someone would need to pay for it (P16) - Once reward system stops, the efforts to improve quality of care also stop (the ‘extra’ also stops) (P16) |
| Penalties | - Participant mixed feelings regarding the use of penalties (P11, P17, P10, P12) - Participants negative feelings regarding the use of penalties (P16) - Availability of benchmarking, getting less or more money based on performance (P11) - Need for better indicators for penalties, based more on outcomes that are important for the patient, and nurses (P11) - Penalties could be issued for inappropriate care, referral, bad communication, transfer of information or delayed care etc.(P10) - System of penalties is introduced in the Netherlands (P16) - Financial penalties have potential to impact care transition, but on policy level (P15) - Lack of financial penalties for community nurses (P13,14) - Internal motivations of staff to provide good quality care is more important than financial penalties (P13,14) |
| Limitations in penalties | - Problems with appointing responsible party (P17, P10) - Financial penalties could have an impact on admission policy, for instance, by admitting healthier patients (P15) - Problems with complexity of the patients (P10) - The effect of penalties is short-lived (P16) - Penalties could be harmful and negatively affect quality of care (P16) - Penalties don’t work (P16) - Problems with measuring the quality of care (P12) - There are always individuals who try to “cheat” the system (P12) |

PXX –ID of a participant (as shown in Table 2: Participants’ characteristics)

| **POLAND** | |
| --- | --- |
| **Organizational challenges** | **Basic findings from the interviews** |
| Coordination of resources | - Need for coordinator (P20, P18, P23, P21, P22) - Need for development of LTC infrastructure & resources (beds, facilities, staff) (P17, P23, P21, P22, P19) - Important role of physiotherapists/rehabilitation (P18, P17, P21, P22) - Coordinator should be linked to community, know environment, the patient etc. (P20, P18, P23, P19) - Important role of primary care and need for stronger involvement (P20, P17, P19) - Need for developing binding procedures/regulations regarding the transition/care coordination (P21, P22, P19) - Important role of care assistants (P19, P17) - Important role of nurses (P20, P19) - Important role of charities and volunteers (P23, P19) - Need for timely provision of LTC (P22, P19) - Need for shortening the waiting time for LTC (P22, P19) - Need for better classification of patients according to needs (considering the patient & environment) (P17, P19) - Need for coordination of financial resources between the health and social system (P23) - Medical staff, specifically general practitioner should not be involved in care coordination due to shortage (P23) - Coordinator could be public health graduate, paramedic or a nurse (P23) - It is important to consider the resource management efficiency in order to deliver effective care to higher number of patients (P23) - Questioning the relevance of care coordinator only in the inpatient settings (P23) - Coordination of resources is the most important (P23) - Need for 24/7 availability of doctors in LTC facilities (P21) - Availability of better medications in hospitals than in LTC (P21) - Need for more (multidisciplinary) staff in LTC facilities (P21) - Regulations regarding the kind of staff in LTC facilities (P21) - Need for addressing multiple aspects at once – medical, psychological, social, spiritual (P21) - Care transition should be coordinated from the beginning till the end (P21) |
| Limitations in coordination | - Limited availability of places in LTC facilities (P23, P22, P17) - Limited availability of LTC staff to provide care at home & LTC facilities (P21, P17, P22) - Lack of binding procedures/regulations regarding the transition (P21, P22, P19) - Lack of coordination between providers (P18, P17, P22) - Long waiting time to access LTC facilities (P20, P22) - Limited involvement of primary care (P17, P21) - Lack of coordination between the health and the social system (P23, P21) - Long waiting times for specialized care (P20) - Lack of transitional care coordinator (P20) - Volunteers may not want to perform caring tasks (P17) - Volunteers’ rotation (P17) - Limited staff in hospitals (P21) - In hospitals focus on medical care only (P21) - Not enough settings helping in care transition (P22) - Insufficient number of social care workers (P22) - Infrastructure of some LTC facilities is not functional, adapted (P22) |
| Communication | - Need for better communication between professionals representing different providers and sectors (P21, P22) - Limited or lack of communication with the patient/family regarding discharge (P21, P19) - Importance of good communication between the providers about patients’ needs (P19) - Need for communication with the family (P19) - Need for active communication between engaged professional groups (P21) - Need for timely communication between sending-receiving setting (P22) - Detailed information provided by hospitals to LTC (P17) - Telephone calls to patients should be introduced (P23) - Short telephone communication between hospital and LTC facility (P23) - Lack of communication with the primary care physicians (P23) - Important role of social care workers in communication between LTC facilities (P21) |
| Limitations in communication | - Limited/very limited communication between providers (P20, P18, P17, P21, P23, P22, P19) - No direct communication between providers (P20, P18) - Limited communication between the staff and patient/family during discharge (P21, P19) - Short telephone communication between hospital and LTC facility (P23) - Lack of communication with the primary care (P23, P19) |
| Transfer of information and patient responsibility | - Need for structured/standardized information exchange, especially between the hospital and primary care (P20, P17, P21) - Transfer of documents done by hospital is more accurate than in primary care (P17) - Nurses should be engaged (P18) - Providers receive documents earlier to prepare LTC setting for the patient (P17) - Complete information provided by the hospitals to LTC (P17) - In hospital social care workers are responsible for transfer of information to LTC facilities (P17) - Need for introduction of online platform to transfer the patient’s information – digitalization (P23) - Transferring full medical information from LTC facility to the hospital (P21) - Information card follows the patient (P21) - Need for timely transfer of information between sending-receiving setting, arranging place (P22) |
| Limitations in transfer of information and patient responsibility | - Patient carrying the information (P20, P17, P23, P21) - Very limited, not-detailed transfer of information (P18, P21, P19) - Limited use of the online platform to transfer the information (P20) - Lack of structured information exchange between hospital and primary care (P20) - Very limited transfer of information (P18) - Lack of direct contact with the other provider (P18) - Outdated transferring of information on paper (P23) - Making regulations regarding the need to prepare discharge letters may further burden limited staff (P23) - Lack of information (to the LTC) regarding the resident/patient admitted to the hospital (P21) - Problem with the transfer of the information card within the hospital (single setting) (P21) - Discharge letters are not standardized (P21) |
| Education and involvement | - Preparing and providing education to the patient and/or caregiver, not only in the hospital but also at home (P20, P18, P17, P19) - Need for provision of multidimensional information/education to the patient and the family (P18, P23, P21, P19) - Providing medical and organizational advice by nurses in primary care settings (P20) - Family’s knowledge and involvement play crucial role (P18, P17) - Availability of the program to educate the family (P18) - Patient and family readiness for the transition (P18) - Patient and family has the right to receive the information in the hospital (P17) - District nurse provide education to the family (P17) - Family is responsible for the patient (P17) - Need for the program directed to informal caregivers and their needs (P21) - Availability of courses educating informal caregivers (P22) - Availability of information in forms of leaflets and instructional videos (P19) |
| Limitations in education and involvement | - Patient/caregiver lack of preparedness (P18, P23) - Patient/caregiver limited knowledge and need for informational support (P23, P21) - Staff not obliged to provide support (P23, P18) - Short, unstructured information provided to the patient & caregivers (P20) - Older patients’ impairment (P17) - Family’s unwillingness to be involved in care (P17) - Lack of coordinator that would inform the patient and the caregiver(P23) - Nurses are not trained to inform the patients about the care in other settings (P23) |
| Training and education of staff | - Need for trainings/education of staff (P20, P18, P21, P22) - Availability of trainings regarding geriatric/LTC (P18) - Availability of trainings for care workers – care assistants for patients with caring needs (P17) - Need for providing additional trainings to the care coordinators (if available) (P23) - Physicians are trained with regard to patient’s information card (P23) |
| Limitations in training and education of staff | - Knowledge of staff regarding the organization of LTC system is limited (P21, P23) - Young medical staff do not poses knowledge about transitional care (P18) - No training for nurses on care provision in other settings (P23) |
| Telemedicine and e-Health | - Possibility to monitor some patient groups (P17, P23, P21, P22, P19) - Availability of telephone consultations, tele-information (P20, P21, P22, P19) - Usefulness of video consultations (P20, P23) - Tele-information enables to access the information instantly and remotely (P21, P22) - Introduction of online platform to transfer the patient’s information (P20) - Need for introduction of online platform to transfer the patient’s information - digitalization (P23) - Usefulness of telephone consultations to provide referrals (P20) - E-health may improve communication among parties (P20) |
| Limitations in telemedicine and e-Health | - Limited use of telemedicine and e-health (P17, P19) - Older adults prefer to avoid using telemedicine (P18) - Limited use of telemedicine and e-health (P17) - Long-term use of telemedicine is not beneficial (P17) - Telemedicine is for physically fit patients (P17) - The use of technologies may use scare resources without actual proof it will work (P23) - Lack of funding for telemedicine, rather episodic (P22) |
| Social care | - Need for proactive engagement and involvement of social care workers (P20, P18, P17, P21, P19) - Social care workers are responsible for preparing documents and communicating with LTC facilities (P18, P17, P21, P19) - Need for social care workers to provide holistic care and support (P17, P23, P21) - Availability of social workers seem to be high (P18) - Need for creating the system of delivering hot meals, socialization and monitoring (P23) - Need for social care workers that prepare the receiving setting and family (P21) |
| Limitations in social care | - Insufficient number of social care workers (P17, P22, P23) - Some social care workers are unresponsive, disorganized (P21) - Patients and caregivers do not know how to access help (P18) - Social care worker tasks are limited to administrative role (P17) - Patient’s income may limit access to social care (P17) - Lack of social coordinator (P23) - Needs of older adults to socialize and thus unnecessary doctors’ visits (P23) - Lack of the person checking suitability of the home for the discharged patient (P23) - Competencies of social care workers are very limited (P22) - Lack of coordination between health and social care (P23) - Limited number of hospitals with social care workers, if social care worker is unavailable, patients need to organize the care by themselves (P19) |
| Supporting informal caregivers | - Need for educational/informational/instrumental support (P21, P18) - Need for the coordinator that would guide the patient and the family throughout the entire process. Provide medical, administrative, legal support (P21, P23) - Need for monitoring patients’/family needs (P18) - Some minor help/advice provided by the doctors (P23) - Social care workers that prepare the receiving setting and family (P21) - Need for respite care services (P21) - Availability of trainings for informal caregivers (P22) |
| Limitations in supporting informal caregivers | - Very limited or even no support to the informal caregivers (P17, P23, P21, P18) - Patients and family need to search for support/information/help by their own (P17, P21, P22, P18) - Supporting caregivers and the patient is not mandatory (P21, P23) - Short, unstructured information for the patient/family (P20) - Lack of system providing respite care (P23) - Providing support is episodic, depending on the funding (P22) |
| **Financial challenges** | **Basic findings from the interviews** |
| Reimbursement | - Need for higher reimbursement of LTC facilities (P17, P21, P22, P23) - Need for competitive/higher salaries for LTC staff (P17, P21, P22, P19) - Need for reimbursement for coordination/coordinator (if available) that is satisfactory (P18, P23, P20) - Important role of charities, NGOs, EU and volunteers in providing financial support (P19, P21, P22 ) - Need for higher number of contracts for nurses providing LTC (P19) - Introducing additional reimbursement for uploading patients’ information on the online platform (P20) - Fundholding in primary care may improve care e.g., shorten waiting list to the specialist (P20) - Help from the government to cover the costs of LTC (P18) - Respondents’ very limited knowledge regarding financing (P18) - Reimbursement for individual patient care should be according to the resources used, not according to disability (P17) - Need for coordination of financial resources between the health and social system (P23) - Perhaps budgets should be used to pay for transitional care? (P23) - Perhaps introducing degressive payment system, financing per person-day in LTC (P23) - Need for financing programs supporting informal caregivers (P21) - Potential solution - development of LTC wards next to hospital - lower reimbursement than hospital (P22) - Financing day care home as a local government (P22) - Need for reimbursement of care homes by NFZ (P22) - Need for additional reimbursement beside per capita payment for family nurses for additional services provided (P19) - Need for additional quality-based payments (P19) |
| Limitations in reimbursement | - The role of out-of-pocket payments for LTC (P18, P17, P21, P22, P19) - Low salaries for LTC staff (P17, P21, P22, P19) - Low reimbursements/underestimated contracts for LTC facilities (P17, P23, P21) - Introducing activity-based payments may affect quality (P23, P22, P19) - Fee-for-service may lead to overtreatment (P23) - Activity based payments for nurses could affect quality of services provided (P19) - Fixed contracts with LTC facilities (P17, P21) - No separate reimbursement for transitional care (P20, P23) - Very low reimbursement for the care coordinator (P20) - Not enough contracts for health staff to provide care at home (P21) - Quality-based reimbursements - Difficulty in measuring quality (P23) - Capitation may lead to overuse of services, unnecessary care transitions to specialized care (P20) - Unwillingness to contribute by commune to LTC costs (P17) - Patient’s income - social care may refuse to help (P17) - LTC facilities receive more money for sicker patients = they are preferred over healthier ones (P17) - Separate reimbursement mechanism for the health and the social system (P23) - Flat-rate payments may lead to longer hospitalizations (P23) - Lack of government funding for telemedicine, rather episodic (P22) - Out-of-pocket payments for telemedicine (P22) - Lack of government funding for courses for caregivers, rather episodic (P22) - Additional funding’s streams may be episodic (P22) - Lack of reimbursement of day care homes from National Health Fund (P22) - Need for satisfactory salary for the care coordinator if such role is introduced (P19) |
| Penalties | - Penalties could be issued for inappropriate care, adverse events, different kind of abuse, misuse, abnormalities or for not fulfilling the contract, unnecessary referrals and lack of vital information in the referrals (P20, P21, P19, P17, P22) - Existence of penalties is a necessity (P22) - Respondent unfamiliar with penalties (P18) - Penalties could be issued for care coordinator, if the role of care coordinator exists (P18) - Lack of penalties regarding transitional care (P23) - Penalties should be constructive (P21) - Penalties should be symbolic (P21) |
| Limitations in penalties | - Penalties could be harmful (P21, P22, P18) - Difficulty in measuring quality of care (P18, P23) - Penalties are sometimes inadequate (P21) - Difficulty in estimating responsibility if something goes wrong (P19) - The use of penalties is questionable (P19) |
| Rewards | - Need for introduction of rewards (P22, P18, P21, P20) - Coordinator (if available) should be eligible for rewards (P18, P21) - Additional activities that improve quality of care should be rewarded (P21) - May improve the care (P20) - Rewards must be satisfactory (P20) - Respondent unfamiliar with rewards (P18) - Rewards do not exist in the hospital (P17) |
| Limitations in rewards | - No rewards available (P21, P22, P17) - Problem with measuring quality of care (P23) - The use of rewards is questionable (P19) |

PXX –ID of a participant (as shown in Table 2: Participants’ characteristics)
